# Insertion of Nanoluc into the Extracellular Loops as a Complementary Method To Establish BRET-Based Binding Assays for GPCRs

**DOI:** 10.1021/acsptsci.2c00162

**Published:** 2022-10-31

**Authors:** Lukas Grätz, Christoph Müller, Andrea Pegoli, Lisa Schindler, Günther Bernhardt, Timo Littmann

**Affiliations:** Institute of Pharmacy, Faculty of Chemistry and Pharmacy, University of Regensburg, Universitätsstrasse 31, D-93053 Regensburg, Germany

**Keywords:** G protein-coupled receptor, bioluminescence resonance energy transfer, ligand binding, assay development, binding kinetics

## Abstract

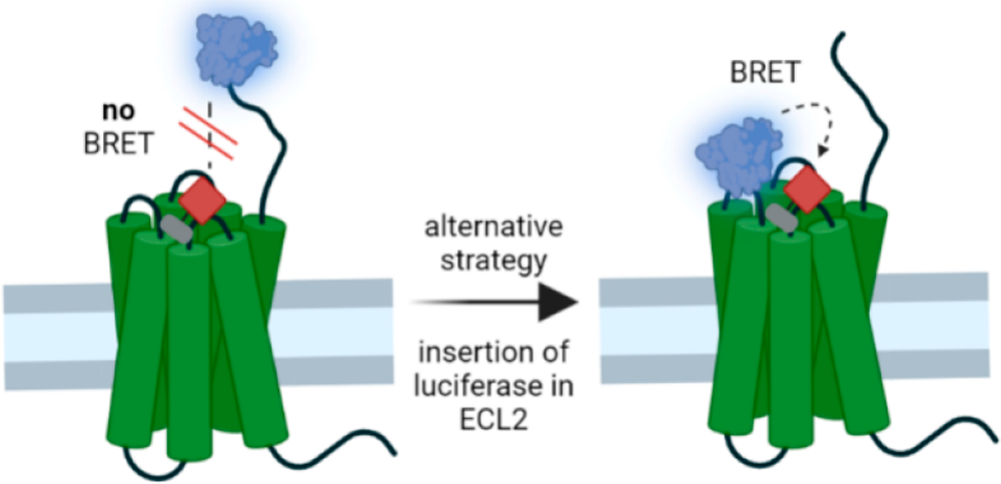

Luminescence-based techniques play an increasingly important role in all areas of biochemical research, including investigations on G protein-coupled receptors (GPCRs). One quite recent and popular addition has been made by introducing bioluminescence resonance energy transfer (BRET)-based binding assays for GPCRs, which are based on the fusion of nanoluciferase (Nluc) to the N-terminus of the receptor and the occurring energy transfer via BRET to a bound fluorescent ligand. However, being based on BRET, the technique is strongly dependent on the distance/orientation between the luciferase and the fluorescent ligand. Here we describe an alternative strategy to establish BRET-based binding assays for GPCRs, where the N-terminal fusion of Nluc did not result in functioning test systems with our fluorescent ligands (e.g., for the neuropeptide Y Y_1_ receptor (Y_1_R) and the neurotensin receptor type 1 (NTS_1_R)). Instead, we introduced Nluc into their second extracellular loop and we obtained binding data for the fluorescent ligands and reported standard ligands (in saturation and competition binding experiments, respectively) comparable to data from the literature. The strategy was transferred to the angiotensin II receptor type 1 (AT_1_R) and the M_1_ muscarinic acetylcholine receptor (M_1_R), which led to affinity estimates comparable to data from radioligand binding experiments. Additionally, an analysis of the binding kinetics of all fluorescent ligands at their respective target was performed using the newly described receptor/Nluc-constructs.

G protein-coupled receptors (GPCRs) represent one of the largest protein families with more than 800 members encoded in the human genome.^1^ All GPCRs share a common architecture, as they all comprise an extracellular N-terminus, an intracellular C-terminus, and seven transmembrane domains, which are connected by three extracellular (ECL1–3) and three intracellular loops (ICL1–3).^2,3^ Due to their abundant expression in humans and their involvement in various (patho)physiological processes, GPCRs represent a very attractive target family in therapy and drug discovery.^4,5^ A mandatory step in the development of novel drug candidates is the assessment of their binding properties to their putative target. In addition to the determination of affinities, investigations on the kinetics of ligand binding are of particular interest.^6−8^ In the last few decades, fluorescence-based techniques emerged as alternative or complementary methods to the widely used radioligand binding assays, as they offer distinct advantages, e.g., in terms of handling, safety, and costs of waste disposal.^9−11^ Especially, proximity-based methods exploiting bioluminescence resonance energy transfer (BRET) or (time-resolved) Förster resonance energy transfer ((TR-)FRET) have gained popularity because of their high-throughput capability, the lower impact of nonspecific binding, and the possibility of performing kinetic measurements in real time without any separation or washing steps.^12−14^ Stoddart et al. introduced a procedure to quantify binding of a fluorescent ligand based on BRET by fusing the very brightly blue light-emitting nanoluciferase (Nluc, λ_max_ ≈ 460 nm)^15^ to the N-terminus of a GPCR to serve as a bioluminescent donor.^16^ Prerequisites for this technique to give robust results are an overlap of the excitation spectrum of the fluorescent ligand (acceptor) with the emission spectrum of the luciferase (donor), an appropriate distance (≈< 10 nm) between donor and acceptor and their correct orientation toward each other.^13,17^ However, although this technique has been used successfully for the determination of binding affinities and binding kinetics at several GPCRs across different classes,^18−23^ we noticed that it was not universally applicable for all ligand–receptor combinations we wanted to assess. Therefore, we aimed at the development of an alternative strategy for those receptor–ligand pairs, for which the N-terminal fusion of Nluc did not result in functioning BRET binding assays.^16^ The neuropeptide Y Y_1_ receptor (Y_1_R) was first taken as a model receptor due to the lack of a specific BRET signal using an N-terminally Nluc-tagged Y_1_R in combination with our fluorescent ligand UR-CM138 (**1**). We examined the insertion of Nluc into unstructured regions within the ECL2 and ECL3 of the Y_1_R, and the former ultimately enabled BRET binding experiments at this receptor. This approach, i.e., insertion of Nluc into the ECL2, was then transferred to the neurotensin receptor type 1 (NTS_1_R), and the applicability was also tested at two receptors with slightly shorter N-termini, the angiotensin II receptor type 1 (AT_1_R) and the M_1_ muscarinic acetylcholine receptor (M_1_R). Besides the determination of the affinities of fluorescently labeled and unlabeled ligands to their target, we performed a detailed analysis of the binding kinetics of the fluorescent ligands with a particular focus on the NTS_1_R and the AT_1_R.

## Results and Discussion

### Search for a Strategy To Establish a BRET Binding Assay at the NPY Y_1_R

**Figure 1:**
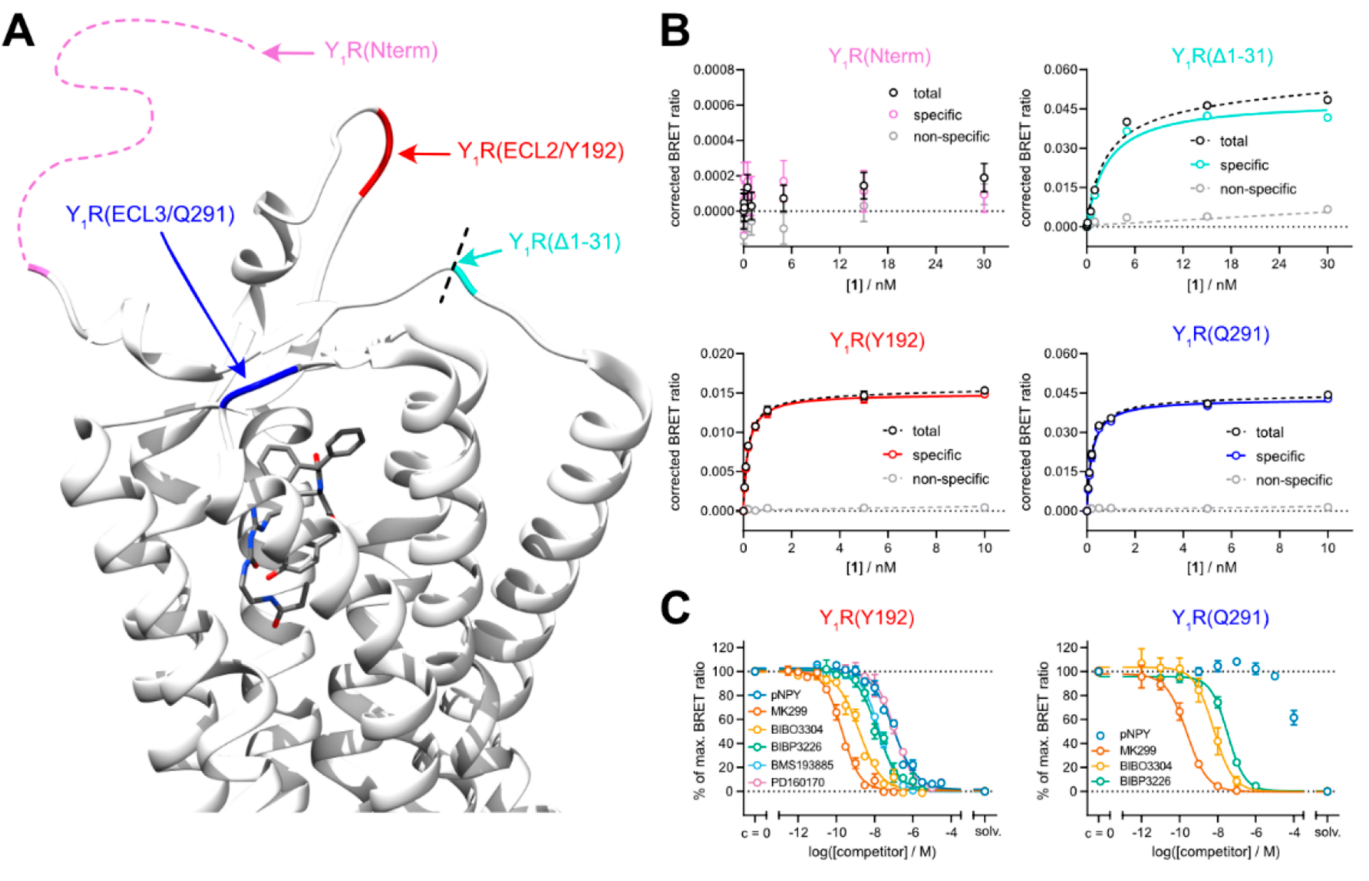
Impact of different attachment and insertion sites of Nluc at the Y_1_R on BRET-based binding. (A) Crystal structure of the Y_1_R in complex with UR-MK299 (PDB-ID: 5ZBQ).^27^ Receptor sites addressed by attachment or insertion of Nluc are indicated by different colors; the N-terminus was artificially extended for the illustration, as it was not completely resolved in the crystal structure. Structure visualization was performed with UCSF Chimera.^28^ (B) Binding isotherms from BRET saturation binding experiments with **1** at HEK293T cells stably expressing the respective Nluc-Y_1_R receptor constructs Nluc-Y_1_R(Nterm), Nluc-Y_1_R(Δ1–31), Nluc-Y_1_R(Y192), or Nluc-Y_1_R(Q291). Nonspecific binding was determined in the presence of BIBO3304 (500-fold excess over the respective concentration of **1**). Data are shown as means ± errors of one representative experiment from a set of three to four independent experiments, each performed in triplicate. Error bars of total and nonspecific binding represent the SEM, the error bars for specific binding represent propagated errors. (C) Displacement curves from BRET competition binding experiments with **1** (*c* = 0.5 nM) and reported Y_1_R ligands at Nluc-Y_1_R(Y192) and Nluc-Y_1_R(Q291), stably expressed in HEK293T cells. Data are shown as means ± SEM of at least three independent experiments, each performed in triplicate. Abbreviation: solv.: solvent control.

**Figure 2:**
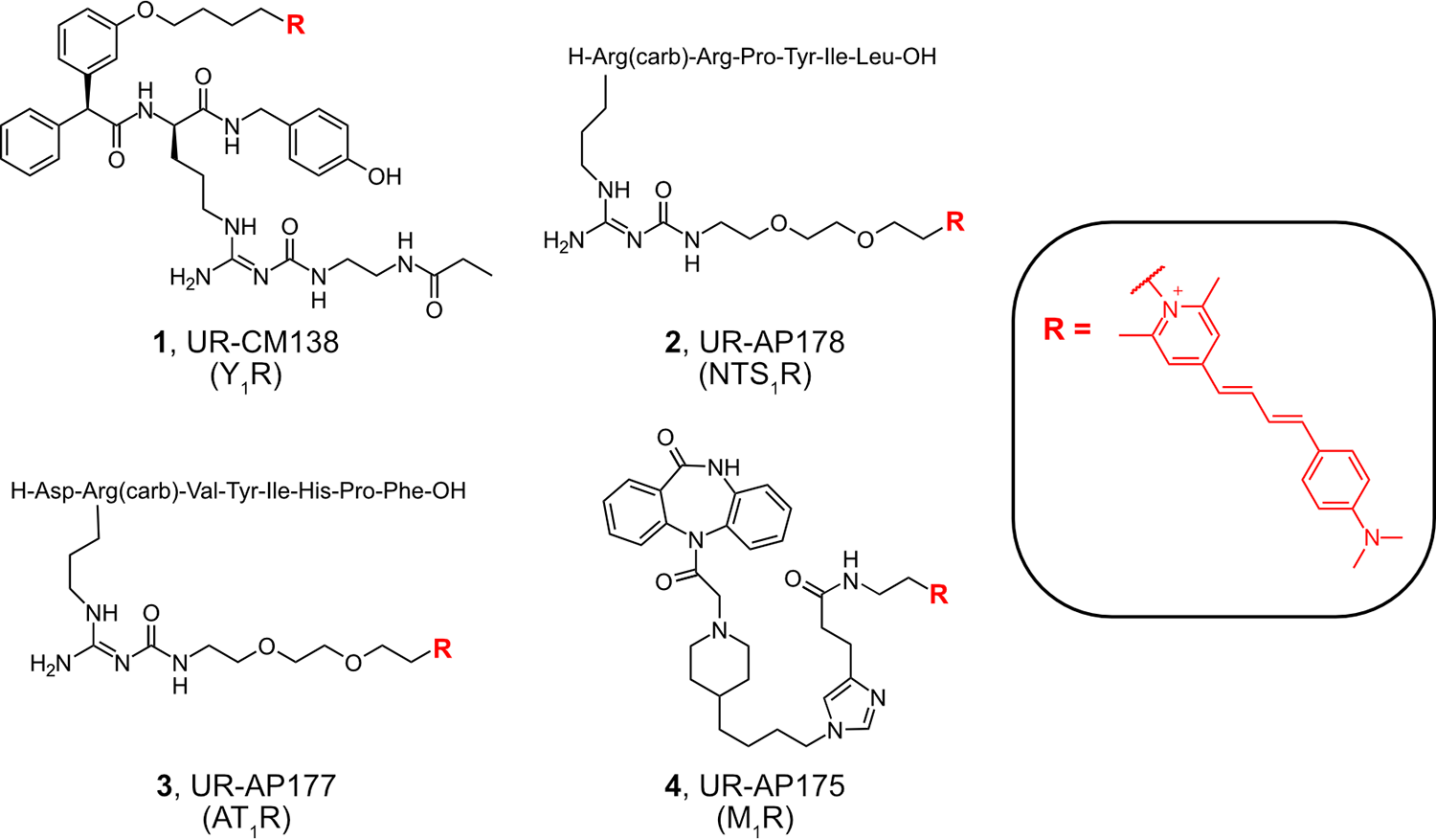
Structures of the investigated fluorescent ligands **1**–**4**.

The approach to establish a BRET binding assay described by Stoddart et al.^16^ was pursued for the Y_1_R, and Nluc was fused to its N-terminus (Nluc-Y_1_R(Nterm)). However, no specific signal was detectable in BRET saturation binding experiments (Figure 1B) with the high-affinity fluorescent Y_1_R ligand UR-CM138^24^ (**1**, see Figure 2). Membrane expression of the tagged receptor could be proven by radioligand saturation binding experiments with [^3^H]UR-MK299^25^ (see Supporting Figure S1A and Supporting Table S1). Furthermore, the retained ability of the radioligand to bind to the modified receptor with high affinity ruled out an abrogation of receptor binding upon fusion with the luciferase. At this point, we hypothesized that shortening the N-terminal domain of the Y_1_R might lead to a higher BRET signal and saturable binding of fluorescent ligand **1**, because the efficiency of BRET is strongly dependent on the distance between donor and acceptor.^13,17^ As a model system to test this hypothesis, we utilized a Y_1_R deletion mutant described by Lindner et al.,^26^ lacking the first 31 amino acids. We removed these same amino acids from the N-terminus and fused Nluc to Asp32 of the Y_1_R via a short linker yielding the construct Nluc-Y_1_R(Δ1–31). Although the ability of this mutant to bind the endogenous agonist neuropeptide Y (NPY) was slightly impaired,^26^ it still represents the mutant that allows the greatest possible proximity between Nluc and the fluorophore. Interestingly, **1** was now able to elicit a specific and saturable signal in a BRET-based saturation binding experiment at the luciferase-tagged truncated Y_1_R (see Figure 1B). However, the determined equilibrium dissociation constant (p*K*_d_ ± SEM (Nluc-Y_1_R(Δ1–31) = 8.54 ± 0.05, cf. Table 1) was found to be inconsistent with the previously obtained results from radioligand competition binding studies at the wild-type Y_1_R (p*K*_i_ = 9.95),^24^ although the radioligand [^3^H]UR-MK299 still showed saturable binding and a high affinity to the truncated and modified receptor (see Supporting Figure S1B and Supporting Table S1).

**Table 1:** Equilibrium Dissociation Constants (p*K*_d_ values) of the Fluorescent Y_1_R Ligand **1** Obtained from BRET Saturation Binding Experiments

compound	receptor (construct)	p*K*_d_ (BRET)^a^	*N*
**1**	Nluc-Y_1_R(Nterm)	n.a.	–
	Nluc-Y_1_R(Δ1–31)	8.54 ± 0.05	3
	Nluc-Y_1_R(Y192)	9.62 ± 0.05	4
	Nluc-Y_1_R(Q291)	9.54 ± 0.07	4

a Determined by BRET saturation binding experiments at intact HEK293T cells stably expressing the respective receptor construct. Data are shown as means ± SEM of *N* independent experiments performed in triplicate. n.a.: not applicable.

In 2018, the structure of the human Y_1_R was published in complex with UR-MK299 (PDB-ID: 5ZBQ),^27^ which represents the parent compound of the used fluorescent ligand **1**. The crystal structure showed that the diphenylacetic acid moiety, which served as the attachment point for the fluorophore in **1** (see Figure 2), is pointing toward the extracellular region of the receptor (see Figure 1A). However, the N-terminus of the Y_1_R is comparably long in size and, even though this region of the receptor was not fully resolved, seems to be pointing away from the ligand binding pocket. This might be an explanation why the N-terminal fusion of Nluc did not yield specific BRET (Figure 1B). The distance could apparently be reduced by truncation of the N-terminus, which resulted in a higher BRET signal, but compromised the binding of **1** to the receptor.

Making use of the structure of the Y_1_R, we pursued a different strategy: to position the luciferase in a more favorable orientation toward the fluorescent ligand, we inserted Nluc into unstructured regions within the second (ECL2) or third extracellular loop (ECL3) of the receptor right after Tyr192 (Nluc-Y_1_R(Y192)) or Gln291 (Nluc-Y_1_R(Q291)), respectively. In BRET saturation binding experiments, **1** showed saturable binding at both Nluc-Y_1_R(Y192) and Nluc-Y_1_R(Q291) (see Figure 1B). However, in contrast to the construct Nluc-Y_1_R(Δ1–31), the equilibrium dissociation constants for **1** (p*K*_d_ ± SEM (Nluc-Y_1_R(Y192)) = 9.62 ± 0.05; p*K*_d_ ± SEM (Nluc-Y_1_R(Q291)) = 9.54 ± 0.07) were both in very good agreement with the results from radioligand competition binding experiments (p*K*_i_ = 9.95).^24^

Interestingly, the signal-to-background ratio obtained for Nluc-Y_1_R(Y192) was comparatively high considering the low number of receptors per cell estimated by radioligand saturation binding experiments (≈ 2500 receptors/cell, see Supporting Figure S1C). This confirms the sensitivity of the BRET-based approach in general, which has already been shown by establishing Nluc-based BRET binding assays for receptors under endogenous promotion (e.g., adenosine A_2B_ receptors) achieved by means of genome editing.^29^

Then BRET competition binding experiments were performed with **1** and different structurally diverse Y_1_R ligands (for structures, see Supporting Figure S2) at Nluc-Y_1_R(Y192) and Nluc-Y_1_R(Q291). The obtained affinities (p*K*_i_ values) of the investigated antagonists (UR-MK299, BIBO3304, BIBP3226, BMS193885, and PD160170) were in very good agreement with data reported in the literature (cf. Table 2 for Nluc-Y_1_R(Y192) and Supporting Table S2 for Nluc-Y_1_R(Q291)). Interestingly, the agonist pNPY (porcine NPY) was not able to displace the fluorescent tracer from Nluc-Y_1_R(Q291) (Figure 1C, right panel). Apparently, the insertion of the luciferase into the ECL3 of the Y_1_R had a negative impact and disrupted the binding of pNPY. In contrast, pNPY was able to displace **1** from Nluc-Y_1_R(Y192) with a p*K*_i_ of 7.49 ± 0.08, which is lower than most reported affinity estimates found in the literature (cf. Table 2). However, it is in very good agreement with the p*K*_i_ value for pNPY determined in flow cytometry-based competition binding experiments using **1** as the fluorescent probe (p*K*_i_ = 7.58), suggesting that the observed discrepancy occurs due to properties of the probe and not the used test system.^24^

**Table 2:** Binding Data (p*K*_i_ Values) of Standard Y_1_R Ligands from BRET Competition Binding Experiments with the Fluorescent Ligand **1** at Nluc-Y_1_R(Y192) Compared to Data from the Literature

compound	p*K*_i_ (BRET)^a^	*N*	reference values (literature)^b^
pNPY	7.49 ± 0.08	5	7.58–9.75^24,25,27,30−32^
UR-MK299	10.20 ± 0.06	6	10.11^25^
BIBO3304	9.31 ± 0.11	5	8.76–9.60^27,30,31^
BIBP3226	8.38 ± 0.11	5	8.14–9.00^25,27,30,33,34^
BMS193885	8.23 ± 0.01	4	7.66–8.48^27,35,36^
PD160170	7.45 ± 0.05	5	7.30,^37^ 7.32^38^

a Determined by BRET competition binding experiments with **1** (*c* = 0.5 nM, *K*_d_ = 0.24 nM) at intact HEK293T cells stably expressing Nluc-Y_1_R(Y192). Data are shown as means ± SEM of *N* independent experiments, each performed in triplicate.

b Reference binding data from the literature, which were obtained from radioactivity-based or fluorescence-based competition binding experiments in different expression systems. Data originate from experiments performed in sodium-containing or sodium-free buffers. For the agonist pNPY, a wide range of p*K*_i_ values has been reported depending on the expression system, the used buffer, and the tracer molecule.

### Transfer of the Novel Strategy to Other GPCRs (NTS_1_R, AT_1_R, M_1_R)

We wanted to investigate if the presented strategy, i.e., the insertion of Nluc into the second extracellular loop of a GPCR to establish BRET binding assays, might be more widely applicable. Therefore, the approach was transferred to the NTS_1_R, which comprises an even longer N-terminus (67 amino acids) than the Y_1_R. Furthermore, the AT_1_R and the M_1_R, which both comprise shorter N-terminal domains, were investigated. Structural information was available for all three receptors,^39−41^ which allowed educated guessing of potential insertion sites by detecting unstructured regions within the extracellular loops and estimating the distance between the luciferase and the binding pocket (for snake plots of all generated constructs, see Supporting Figure S3). The NT(8–13) (neurotensin (8–13))-based NTS_1_R ligand UR-AP178 (**2**),^42^ the angiotensin II-derived AT_1_R ligand UR-AP177 (**3**), and the MR ligand UR-AP175 (**4**)^43^ were used as fluorescent ligands (see Figure 2). All tested ligands carried the fluorescence label Py-5, as it was previously shown to be ideally suited for a combination with Nluc in BRET binding assays.^18^

**Figure 3:**
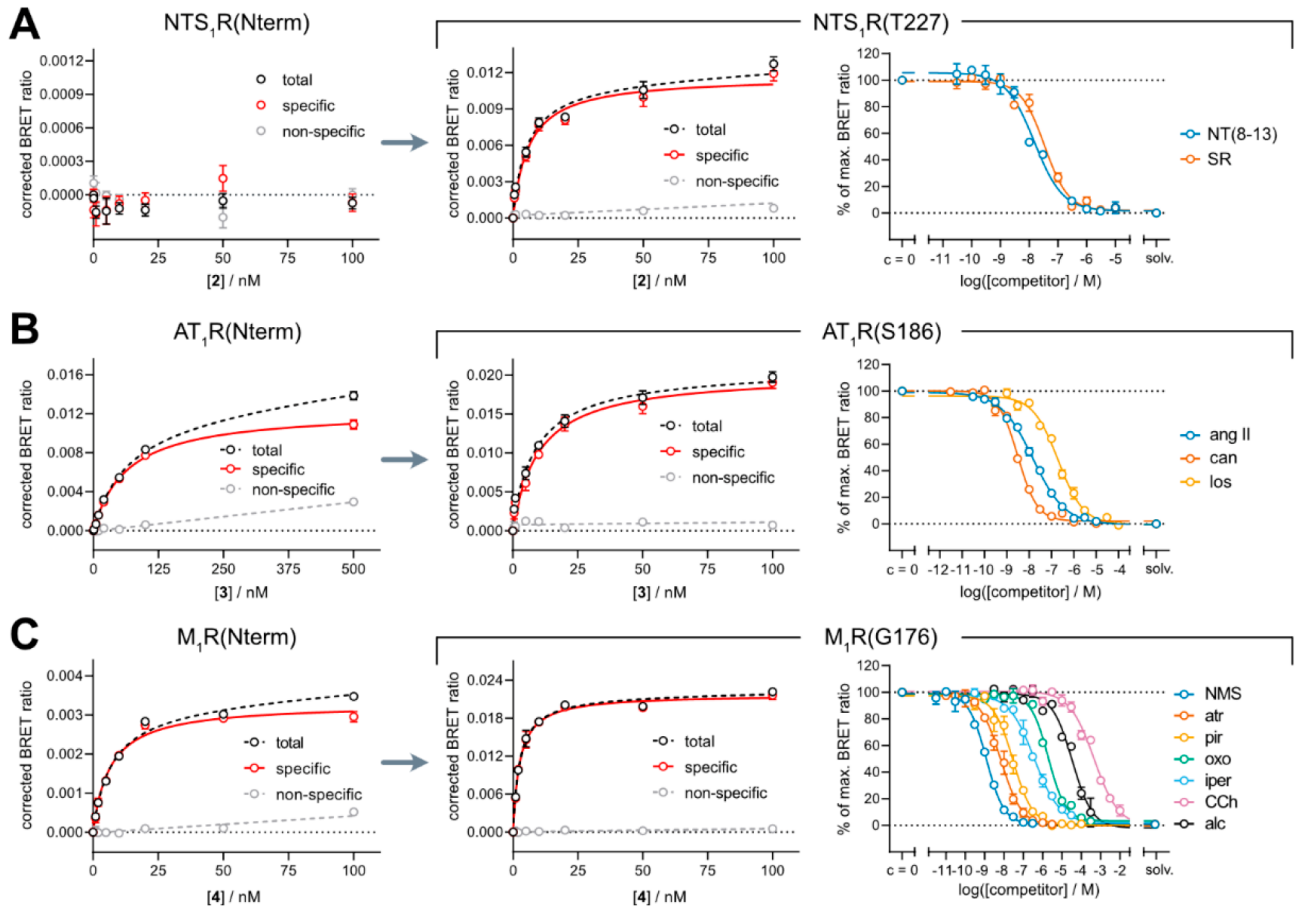
BRET binding data at the NTS_1_R (A), the AT_1_R (B), and the M_1_R (C). Shown are binding isotherms from saturation binding experiments with the fluorescent ligands **2** (A), **3** (B), or **4** (C) at HEK293T cells stably expressing the N-terminally Nluc-tagged receptors (Nluc-NTS_1_R(Nterm) in A, Nluc-AT_1_R(Nterm) in B, or Nluc-M_1_R(Nterm) in C) or the respective receptor constructs with Nluc inserted into the second extracellular loop (Nluc-NTS_1_R(T227) in A, Nluc-AT_1_R(S186) in B, or Nluc-M_1_R(G176) in C). Nonspecific binding was assessed in the presence of an excess of SR142948 (A, 100-fold excess), candesartan (B, 100-fold excess) or atropine (C, 500-fold excess) (for all experiments: excess over the concentration of the fluorescent ligand). Data are shown as means ± errors of one representative experiment from a set of at least three independent experiments, each performed in triplicate. Error bars of total and nonspecific binding represent the SEM, whereas error bars of specific binding represent propagated errors. In the right section of the figure, displacement curves from BRET competition binding experiments with the fluorescent ligands **2** (A, *c* = 5 nM), **3** (B, *c* = 10 nM) or **4** (C, *c* = 5 nM) and corresponding standard ligands at the respective receptor construct, stably expressed in HEK293T cells, are shown. Data are shown as means ± SEM of at least four independent experiments, each performed in triplicate. Abbreviations: solv.: solvent control; NT(8–13): neurotensin (8–13); SR: SR142948; ang II: angiotensin II; can: candesartan; los: losartan; NMS: *N*-methylscopolamine; atr: atropine; pir: pirenzepine; oxo: oxotremorine; iper: iperoxo; CCh: carbachol; alc: alcuronium.

By analogy with the results for the Y_1_R, no specific binding of **2** was observed in BRET saturation binding experiments at the N-terminally Nluc-tagged NTS_1_ receptor (Nluc-NTS_1_R(Nterm), see Figure 3A). Again, this observation suggested that the length and orientation of the N-terminus of a given receptor with respect to the fluorescent ligand might play an important role in whether the N-terminal fusion of Nluc^16^ results in a functioning BRET binding assay. According to our novel strategy, Nluc was inserted into the ECL2 of the NTS_1_R downstream of Thr227 (Nluc-NTS_1_R(T227)). Membrane localization and retained binding properties of the receptor–luciferase fusion protein were confirmed by radioligand saturation binding. The radioligand [^3^H]UR-MK300^44^ bound to the modified NTS_1_ receptor in a saturable manner with an affinity not more than half a log unit below the p*K*_d_ values obtained at unmodified receptors (see Supporting Figure S1D and Supporting Table S1).^44^ In contrast to Nluc-NTS_1_R(Nterm), a specific BRET signal could be observed in BRET saturation binding experiments with **2** at Nluc-NTS_1_R(T227) (see Figure 3A). The p*K*_d_ value for **2** was found to be comparable to the literature-described value (p*K*_d_ ± SEM (Nluc-NTS_1_R(T227)) = 8.32 ± 0.08).^42^ Similarly, BRET competition binding experiments with **2** yielded p*K*_i_ values for the reference agonist NT(8–13) and the antagonist SR142948 (for structures, see Supporting Figure S2) with no more than half an order of magnitude difference from data described in the literature (cf. Table 4). Moreover, Nluc-NTS_1_R(T227) was still functional in a Fura-2 Ca^2+^ assay (see Supporting Figure S4A).

Next, we extended our approach to the AT_1_R and the M_1_R, both comprising shorter N-termini. As a BRET binding assay for the N-terminally Nluc-tagged AT_1_R (Nluc-AT_1_R(Nterm)) had been reported,^16^ we expected that BRET saturation binding experiments with the angiotensin II-derived ligand **3** would also yield a concentration-dependent and saturable specific BRET signal (see Figure 3B). Surprisingly, the p*K*_d_ value for **3** at Nluc-AT_1_R(Nterm) (p*K*_d_ ± SEM = 7.24 ± 0.07) was not comparable to results from radioligand competition binding experiments (p*K*_i_ ± SD = 8.69 ± 0.05, for displacement curve, see Supporting Figure S5) at untagged receptors. As the ECL2 was the most convincing insertion site for both the Y_1_R and the NTS_1_R, we followed this approach for the AT_1_R as well by introducing Nluc downstream of Ser186 (Nluc-AT_1_R(S186)). The generated receptor–luciferase fusion protein was still capable of binding the AT_1_R radioligand [^3^H]UR-MK292^44^ (see Supporting Figure S1E) and was functionally active in a Fura-2 Ca^2+^ assay (see Supporting Figure S4B). The fluorescent ligand **3** showed saturable binding to the modified receptor but, in comparison to the N-terminally tagged variant,^16^ bound with higher affinity (p*K*_d_ ± SEM (Nluc-AT_1_R(S186)) = 8.04 ± 0.08). Although being more in line, the obtained p*K*_d_ value was still slightly lower than the results from radioligand competition binding experiments. However, the CHO cells used for the radioligand competition binding experiments were stably cotransfected with the Gα_16_ subunit, which stabilizes the present AT_1_Rs in an active receptor conformation, thus favoring agonist binding.^45^ This presumably led to a higher affinity estimate for **3**, a compound derived from the endogenous AT_1_R agonist angiotensin II. The same explanation also holds true for the discrepancy between the p*K*_d_ value of the radiolabeled agonist [^3^H]UR-MK292^44^ from radioligand saturation binding experiments at Nluc-AT_1_R(S186) and the previously reported results from experiments at CHO-AT_1_R cells stably coexpressing Gα_16_ (see Supporting Figure S1E and Supporting Table S1).^44^ BRET competition binding experiments with **3** and the agonist angiotensin II or the antagonists candesartan and losartan (Figure 3B, right panel; structures see Supporting Figure S2) resulted in p*K*_i_ values in very good agreement with previously reported radioligand competition binding data (cf. Table 4), especially when compared with affinities determined at cells devoid of the stably coexpressed Gα_16_ subunit.^46−49^

BRET saturation binding experiments with the fluorescent MR ligand **4** at the N-terminally Nluc-tagged M_1_ receptor (Nluc-M_1_R(Nterm)) resulted in a specific BRET signal (Figure 3C) and a p*K*_d_ value of 8.22 ± 0.06 (cf. Table 3), which was comparable, although slightly lower than results from radioligand competition binding experiments.^43^ Applying our novel strategy, Nluc was inserted into the ECL2 of the receptor right after Gly176 (Nluc-M_1_R(G176)). Compared to the results at Nluc-M_1_R(Nterm), a clear increase in signal-to-background ratio (see Figure 3C) was observed. At the same time, the p*K*_d_ value originating from BRET saturation binding experiments at Nluc-M_1_R(G176) (p*K*_d_ ± SEM = 8.65 ± 0.04) matched well with the results at Nluc-M_1_R(Nterm) and even better with the p*K*_i_ value from radioligand competition binding experiments at untagged M_1_ receptors.^43^ Both of these observations, i.e., the increase in signal-to-background ratio by inserting Nluc into the ECL2 and the maintained p*K*_d_/p*K*_i_ values, could also be observed for the TAMRA-labeled MR ligand UR-CG072^43^ (**6**) and the Py-1-labeled MR ligand UR-CG074^43^ (**7**, see Supporting Figure S6 and Supporting Table S3), both based on the same precursor.

**Table 3:** Equilibrium Dissociation Constants (p*K*_d_ values) of the Investigated Fluorescent Ligands **2**, **3**, and **4** Obtained from BRET Saturation Binding Experiments

compound	receptor construct	p*K*_d_ (BRET)^a^	*N*
**2**	Nluc-NTS_1_R(Nterm)	n.a.	–
	Nluc-NTS_1_R(T227)	8.32 ± 0.08	4
**3**	Nluc-AT_1_R(Nterm)	7.24 ± 0.07	3
	Nluc-AT_1_R(S186)	8.04 ± 0.08	5
**4**	Nluc-M_1_R(Nterm)	8.22 ± 0.06	5
	Nluc-M_1_R(G176)	8.65 ± 0.04	4

a Determined by BRET saturation binding experiments at intact HEK293T cells stably expressing the indicated receptor construct. Data are shown as means ± SEM of *N* independent experiments performed in triplicate.

**Table 4:** Binding Data (p*K*_i_ values) of Standard NTS_1_R, AT_1_R, and M_1_R Ligands from BRET Competition Binding Experiments at the Indicated Receptor Construct Using **2** (Nluc-NTS_1_R(T227)), **3** (Nluc-AT_1_R(S186)), or **4** (Nluc-M_1_R(G176)) as the Fluorescent Probe

receptor construct	compound	p*K*_i_ (BRET)^a^	*N*	reference values (literature)^b^
Nluc-NTS_1_R(T227)	NT(8–13)	8.24 ± 0.16	4	8.27–9.85^42,44,50−52^
	SR142948	7.93 ± 0.12	4	8.05–8.99^42,44,50,53^
Nluc-AT_1_R(S186)	angiotensin II	8.15 ± 0.07	5	7.61–9.62^44,46−49^
	candesartan	8.82 ± 0.04	5	8.46–10.28^41,44,46−48^
	losartan	7.06 ± 0.05	4	7.23–8.00^41,44,46,48,49^
Nluc-M_1_R(G176)	carbachol	3.89 ± 0.09	6	3.46–4.52^54−59^
	oxotremorine	6.00 ± 0.11	5	5.48–5.86^55,57,59^
	iperoxo	7.00 ± 0.07	6	6.46^56^
	atropine	8.60 ± 0.09	6	8.50–9.70^55,60−65^
	NMS	9.41 ± 0.03	5	9.49–10.22^55,62,66^
	pirenzepine	7.91 ± 0.10	5	6.85–8.29^55,60,63,64,67^
	alcuronium	4.99 ± 0.08	4	5.01,^57^ 5.25^55^

a Determined by BRET competition binding experiments with **2** (Nluc-NTS_1_R(T227), *c* = 5 nM, *K*_d_ = 4.82 nM), **3** (Nluc-AT_1_R(S186), *c* = 10 nM, *K*_d_ = 9.02 nM), or **4** (Nluc-M_1_R(G176), *c* = 5 nM, *K*_d_ = 2.26 nM) at intact HEK293T cells stably expressing the indicated receptor construct. Data are shown as means ± SEM of *N* independent experiments, each performed in triplicate.

b Reference binding data from literature determined by radioactivity-based or fluorescence-based competition binding experiments in different expression systems.

BRET competition binding experiments at the Nluc-M_1_R(G176) with **4** and several reference ligands yielded p*K*_i_ values in good agreement with reported data from the literature (cf. Table 4). Even the allosteric modulator alcuronium could still bind to the modified receptor and displace the dualsteric ligand **4** completely from Nluc-M_1_R(G176).

### Investigations on the Binding Kinetics at the Generated Constructs

**Figure 4:**
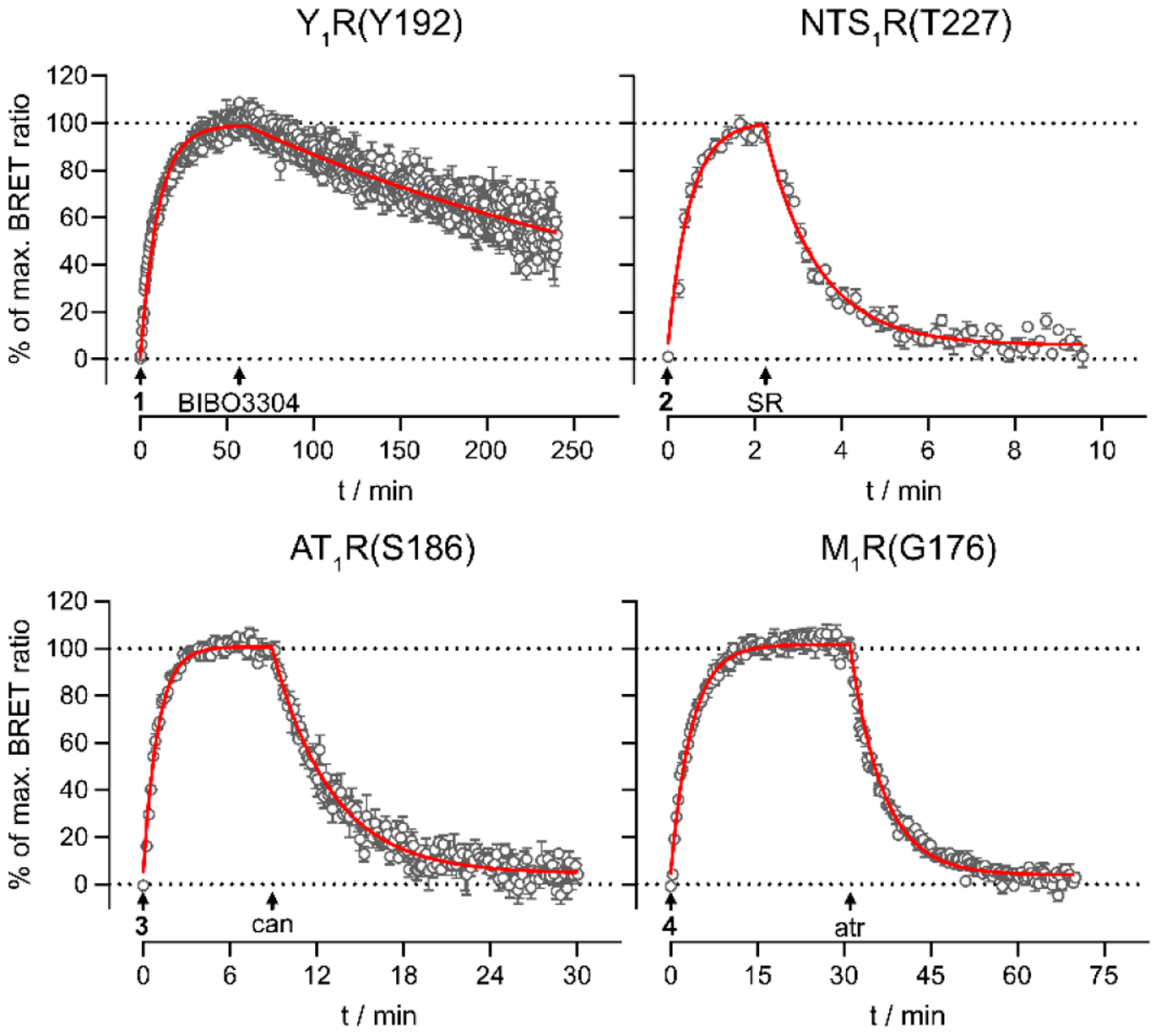
Association and dissociation kinetics (specific binding) of the fluorescent ligands **1** (Nluc-Y_1_R(Y192), *c* = 0.5 nM), **2** (Nluc-NTS_1_R(T227)), *c* = 10 nM), **3** (Nluc-AT_1_R(S186), *c* = 10 nM), and **4** (Nluc-M_1_R(G176), *c* = 5 nM) from kinetic BRET binding experiments at intact HEK293T cells stably expressing the respective constructs (note the differing time scales). Association was initiated at *t* = 0 min by the addition of the respective fluorescent ligand. Dissociation was initiated at the indicated time points by the addition of BIBO3304 (Nluc-Y_1_R(Y192), *c* = 500 nM), SR142948 (Nluc-NTS_1_R(T227)), *c* = 2.5 μM), candesartan (Nluc-AT_1_R(S186), *c* = 2.5 μM), or atropine (Nluc-M_1_R(G176), *c* = 5 μM). Data are shown as means ± propagated errors and are representative of three independent experiments, each performed in triplicate. Abbreviations: SR: SR142948; can: candesartan; atr: atropine.

One of the most useful characteristics of the BRET binding assay is the possibility to record the association and dissociation of fluorescent ligands in real time. Therefore, kinetic BRET binding experiments were conducted to obtain more information about the binding behavior of the fluorescent ligands at their target receptor/Nluc fusion proteins (see Figure 4 and Table 5).

**Table 5:** Kinetic Constants of the Fluorescent Ligands **1**–**4** Determined in BRET-Based Binding Experiments

receptor construct	compound	*k*_off_ [min^–1^]^a^	*k*_on_ [nM^–1^ min^–1^]^b^	p*K*_d_^kinetic,^^c^	p*K*_d_^equilibrium,^^d^
Nluc-Y_1_R (Y192)	**1**	0.004 ± 0.001	0.143 ± 0.005	10.62 ± 0.07	9.62 ± 0.05
Nluc-NTS_1_R(T227)	**2**	0.830 ± 0.051	0.120 ± 0.012	8.16 ± 0.07	8.32 ± 0.08
Nluc-AT_1_R(S186)	**3**	0.236 ± 0.008	0.077 ± 0.005	8.51 ± 0.04	8.04 ± 0.08
Nluc-M_1_R(G176)	**4**	0.178 ± 0.002	0.022 ± 0.007	8.04 ± 0.15	8.65 ± 0.04

a Dissociation rate constant (*k*_off_). Determined by kinetic BRET binding experiments with **1** (*c* = 0.5 nM), **2** (*c* = 10 nM), **3** (*c* = 10 nM) and **4** (*c* = 5 nM) at intact HEK293T cells stably expressing the indicated receptor construct. Data represent the means ± SEM of three independent experiments performed in triplicate.

b Association rate constant (*k*_on_). Determined by kinetic BRET binding experiments with **1** (*c* = 0.5 nM), **2** (*c* = 10 nM), **3** (*c* = 10 nM) and **4** (*c* = 5 nM) at intact HEK293T cells stably expressing the indicated receptor construct. Data represent the means ± SEM of three independent experiments performed in triplicate.

c Kinetically derived dissociation constant (*K*_d_^kinetic^), which was transformed into the p*K*_d_ value for each independent experiment; indicated values represent means ± SEM of the p*K*_d_^kinetic^ values. Determined by kinetic BRET binding experiments with **1** (*c* = 0.5 nM), **2** (*c* = 10 nM), **3** (*c* = 10 nM) and **4** (*c* = 5 nM) at intact HEK293T cells stably expressing the indicated receptor construct. Data represent the means ± SEM of three independent experiments performed in triplicate.

d Equilibrium dissociation constants from BRET saturation binding experiments; values were taken from Table 1 and Table 3, respectively, and renamed as p*K*_d_^equilibrium^ for clarification.

The fluorescent Y_1_R ligand **1** (*c* = 0.5 nM) showed an association to Nluc-Y_1_R(Y192) within 60 min and very slow dissociation kinetics (≈ 40% still bound after 4 h). The slow dissociation kinetics also contribute to the deviation of the kinetically derived dissociation constant (p*K*_d_^kinetic^) from the results from equilibrium experiments (cf. Table 5). However, as described above, the slow dissociation of the fluorescent tracer from the receptor did not preclude the complete displacement of the fluorescent ligand in competition binding experiments and p*K*_i_ values in good agreement with data from the literature (cf. Table 2). Association of the fluorescent NTS_1_R ligand **2** (*c* = 10 nM) occurred rapidly within 2 min, and the ligand could be displaced completely from Nluc-NTS_1_R(T227) within 10 min (see Figure 4). This observation was consistent with previous results from confocal microscopy experiments with similar fluorescent ligands based on the same pharmacophore.^42^ The fluorescent ligands **3** (*c* = 10 nM) and **4** (*c* = 5 nM) showed a moderate association rate to their respective targets. After addition of a competitive ligand, both compounds could be displaced completely from their receptors within the observed time period (see Figure 4). The determined p*K*_d_^kinetic^ values were matching the respective p*K*_d_ values from equilibrium saturation binding experiments.

**Figure 5:**
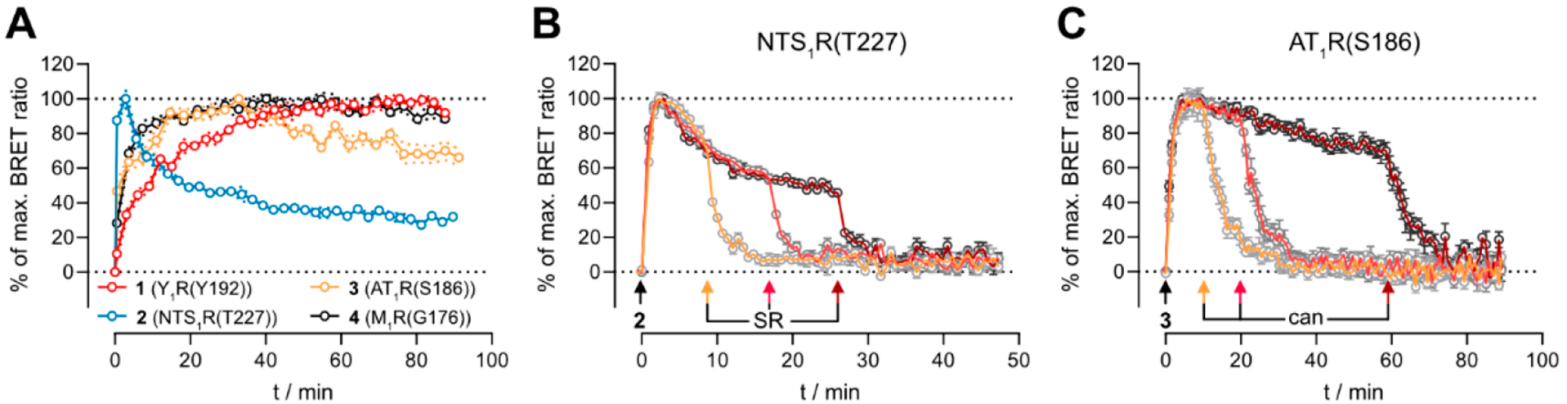
(A) Comparison of the association kinetics (specific binding) of the fluorescent ligands **1** (Nluc-Y_1_R(Y192), *c* = 0.5 nM), **2** (Nluc-NTS_1_R(T227), *c* = 5 nM), **3** (Nluc-AT_1_R(S186), *c* = 5 nM), and **4** (Nluc-M_1_R(G176), *c* = 5 nM) from BRET-based binding experiments at intact HEK293T cells stably expressing the respective construct. (B, C) Dissociation of **2** (*c* = 10 nM) from Nluc-NTS_1_R(T227) (B) or **3** (*c* = 10 nM) from Nluc-AT_1_R(S186) (C). The fluorescent ligands were added at the time point *t* = 0 min. Dissociation was initiated after different time points (indicated by differently colored arrows) by the addition of SR142948 (B, *c* = 2.5 μM) or candesartan (C, *c* = 2.5 μM). Data are shown as means ± propagated errors. Data shown are representative of at least three independent experiments, each performed in triplicate. Data in A were sampled from kinetic saturation binding experiments performed at the respective construct.

Interestingly, the association kinetics of ligands **2** and **3** to their respective target showed a peak followed by a decrease in signal without the addition of a competitive ligand when observing them for a longer time. In contrast, the kinetic traces of the antagonists **1** at Nluc-Y_1_R(Y192) and **4** at Nluc-M_1_R(G176) both reached a stable plateau (see Figure 5A). The qualitative curve shapes were independent of the ligand concentration used (see Supporting Figure S7 for exemplary kinetic traces of BRET saturation binding experiments). Similar observations have been previously reported for BRET binding assays at the receptor tyrosine kinase VEGFR2 and were explained by agonist-dependent internalization of the receptor and subsequent dissociation of the ligand–receptor complex.^68,69^ Furthermore, immunostaining studies at the NTS_1_R and the AT_1_R indicated that agonist binding can cause internalization and that the ligand and the receptor are then localized in different compartments within the cells.^70,71^ As the fluorescent ligands used are both putative agonists at their target, we assumed that this uncoupling of ligand and receptor after internalization was responsible for the observed signal decay in our BRET-based binding assay. To investigate this assumption, BRET saturation binding experiments were performed at cell homogenates of HEK293T cells stably expressing Nluc-NTS_1_R(T227) or Nluc-AT_1_R(S186), as receptor internalization cannot occur in cell homogenates. The fluorescent ligands **2** and **3** bound in a saturable manner to the homogenates, and after association, the BRET signal was stable over a longer period of time (see Supporting Figure S8). This corroborated our assumption that the signal decay shown in Figure 5A was indeed caused by internalization processes. It should be noted that the determined p*K*_d_ values for **2** and **3** at the cell homogenates differed by around two log units from the results obtained using intact cells (cf. Supporting Table S4), presumably due to the uncoupling of the heterotrimeric G protein from the receptor in homogenates, which consequently results in a lower agonist affinity to the free receptor.^72,73^

The aforementioned experiments suggested the dissociation of receptor and fluorescent ligand after internalization. We hypothesized that it should be possible to abolish the residual BRET signal at any given time point by addition of a competitor, because the residual BRET should only originate from fluorescent ligands bound to receptors at the cell surface. Therefore, further kinetic BRET binding experiments were performed by analogy with the experiments depicted in Figure 4, with the following modification: dissociation of the fluorescent ligands was initiated after different time points by addition of a competitive ligand, when the BRET signal had already started to decrease (Figure 5B and 5C). Independent of the time point of dissociation initiation, both fluorescent ligands could be displaced completely from Nluc-NTS_1_R(T227) or Nluc-AT_1_R(S186) with similar dissociation kinetics. Therefore, we conclude that the detected BRET signal originates from ligand-bound receptors at the membrane and not from internalized receptors.

As expected, following the time course of BRET competition binding experiments at Nluc-NTS_1_R(T227) and Nluc-AT_1_R(S186) with **2** and **3**, respectively, resulted in similar kinetic traces (see Supporting Figure S9). However, despite the steady signal decrease over time, the pIC_50_ values of the investigated competitive ligands determined at different time points stabilized quickly (maximum: 45 min for losartan and angiotensin II) (see Supporting Figures S9F and S9G), suggesting equilibrium.

## Conclusion

Here we present a complementary approach to establish BRET binding assays for GPCRs by inserting the bioluminescent donor Nluc into the ECL2 of the receptor instead of fusing it to the N-terminus. This strategy proved especially useful for class A GPCRs with slightly longer N-termini (exceeding 40 amino acids), e.g., the Y_1_R and the NTS_1_R, as BRET-based binding assays with a specific signal and retained affinity of our fluorescent ligands were only possible with the herein presented approach.

It should however be noted that it is not only the length of the N-terminal domain, which decides whether the N-terminal fusion of Nluc results in a functioning BRET binding assay. The used fluorescent ligand also plays a substantial role. For example, a BRET-based binding assay with an N-terminally Nluc-tagged Y_1_R has recently been described in combination with a TAMRA-labeled pNPY as the fluorescent ligand,^74^ even though this approach did not work robustly with our labeled ligand UR-CM138 (**1**). Moreover, BRET binding assays have been described for receptors with substantially larger N-terminal domains than the receptors tested in this study, especially for receptors of the class F.^22,75^ Therefore, the combination of fluorescent ligand and receptor seems to be the main determinant whether the N-terminal fusion of Nluc results in a functioning binding assay.

Our strategy, i.e., insertion of Nluc into the ECL2 of a GPCR was also applicable to receptors with shorter N-terminal domains. For those receptors, compared to the N-terminal fusion of Nluc, our approach resulted in an affinity estimate for the fluorescent ligand comparable or even more in line with literature. BRET competition binding experiments at all receptor constructs yielded affinity values comparable to literature-described data for several standard ligands. Kinetic binding experiments with the agonistic ligands **2** and **3** at Nluc-NTS_1_R(T227) and Nluc-AT_1_R(S186), respectively, resulted in association curves, which did not end in plateaus, but showed a decline after a peak was reached. This could be explained by receptor internalization, occurring in live cells, as plateau-reaching association curves could be obtained by BRET saturation binding experiments with **2** and **3** at cell homogenates.

Taken together, the herein presented strategy, i.e., insertion of Nluc into the ECL2 of a GPCR, will be of high value for the establishment of BRET binding assays and represents an alternative approach in particular but not exclusively for receptors, for which an N-terminal Nluc fusion delivers no or insufficient BRET upon addition of a fluorescent ligand.

## Experimental Section

### Materials

Dulbecco’s Modified Eagle’s Medium (DMEM), l-glutamine, fetal calf serum (FCS), HEPES, and Triton X-100 were from Sigma-Aldrich (Munich, Germany). Trypsin/EDTA (0.05%/0.02%) was from Biochrom (Berlin, Germany). Leibovitz’s L-15 medium (L-15) and geneticin (G418) were from Fisher Scientific (Nidderau, Germany). Bovine serum albumin (BSA) and bacitracin were from SERVA Electrophoresis (Heidelberg, Germany). Furimazine (Nano-Glo Live Cell Substrate) was purchased from Promega (Mannheim, Germany). The pcDNA3.1 vector was from Thermo Fisher (Nidderau, Germany).

Alcuronium chloride (alc), atropine sulfate (atr), carbachol (CCh), iperoxo iodide (iper), *N*-methyl scopolamine bromide (NMS), and pirenzepine dihydrochloride (pir) were from Sigma-Aldrich (Munich, Germany). BMS193885, oxotremorine sesquifumarate (oxo), PD160170, and SR142948 (SR) were from Tocris Bioscience (Bristol, UK). Candesartan (can) and losartan potassium salt (los) were kindly provided by Hexal AG (Holzkirchen, Germany). Neurotensin(8–13) (NT(8–13)) and porcine neuropeptide Y (pNPY) were from SynPeptide (Shanghai, China), whereas angiotensin II (ang II) was from Bachem (Bubendorf, Switzerland). The radioligand [^3^H]NMS (specific activity = 80 Ci/mmol) was purchased from American Radiolabeled Chemicals Inc. (St. Louis, MO). The syntheses of UR-MK299 and the radioligand [^3^H]UR-MK299^25^ as well as the syntheses of the radioligands [^3^H]UR-MK292 and [^3^H]UR-MK300 were reported elsewhere.^44^ The syntheses of the fluorescent ligands UR-CM138 (**1**,Y_1_R), UR-AP178 (**2**, NTS_1_R), and UR-AP175 (**4**, M_1_R) are described elsewhere.^24,42,43^ Syntheses of the fluorescent AT_1_R ligand UR-AP177 (**3**) and the Y_1_R ligand BIBP3226 can be found in the Supporting Information. Stock solutions of the fluorescent ligands were prepared in DMSO and stored in aliquots at −80 °C. Stock solutions of angiotensin II and NT(8–13) were prepared in a mixture of ethanol and 50 mM HCl (30:70). Stock solutions of the other standard ligands were prepared in H_2_O or in DMSO, whenever the compound was insoluble in H_2_O.

### Generation of Plasmids

Plasmids containing the sequences of the investigated human GPCRs (neuropeptide Y Y_1_ receptor (Y_1_R), neurotensin receptor type 1 (NTS_1_R), angiotensin II receptor type 1 (AT_1_R), and M_1_ muscarinic acetylcholine receptor (M_1_R)) were purchased from the cDNA Resource Center (Rolla, MO). The vector encoding the nanoluciferase (Nluc) was obtained from Promega (Mannheim, Germany). All constructs in this study were generated using standard PCR and restriction techniques. The vectors encoding the N-terminally Nluc-tagged receptors (Nterm) were prepared by exchanging the receptor sequence in the previously described pcDNA3.1 NLuc-hH_4_R^18^ with the respective GPCR of interest. The pcDNA3.1 Nluc-Y_1_R(Δ1–31) encoding Nluc fused N-terminally to a truncated Y_1_ receptor (lacking amino acids 1–31) was obtained analogously. For the constructs with the luciferase being located within the ECLs, Nluc was integrated into the receptor sequence downstream of the indicated amino acids (e.g., pcDNA3.1 Nluc-Y_1_R(Y192): after the tyrosine in position 192). A short flexible linker sequence consisting of Gly and Ser was used to connect the luciferase to the receptor at both the 5′ and 3′ ends. The sequence of all generated plasmids was verified by sequencing (Eurofins Genomics, Ebersberg, Germany).

### Cell Culture and Generation of Stable Transfectants

HEK293T cells (kind gift from Prof. Dr. Wulf Schneider, Institute for Medical Microbiology and Hygiene, University of Regensburg, Germany) were cultivated in DMEM supplemented with 2 mM l-glutamine and 10% FCS at 37 °C in a water-saturated atmosphere (containing 5% CO_2_). One day before transfection, the cells were seeded at a density of 3 × 10^5^ cells/mL in six-well plates (Sarstedt, Nümbrecht, Germany). On the following day, cells were transfected with 2 μg of the respective cDNA using X-tremeGENE HP (Roche Diagnostics, Mannheim, Germany) as the transfection reagent (used according to manufacturer’s protocol). Stably transfected cells were selected with 1 mg/mL G418, while cultivation was continued with 600 μg/mL G418. All cells were regularly tested for mycoplasma infection using the Venor GeM Mycoplasma Detection Kit (Minerva Biolabs, Berlin, Germany) and were negative.

### Radioligand Binding Experiments

Radioligand saturation binding experiments at intact, suspended HEK293T cells stably expressing Nluc-Y_1_R(Nterm), Nluc-Y_1_R(Δ1–31), Nluc-Y_1_R(Y192), Nluc-M_1_R(Nterm), or Nluc-M_1_R(G176) were performed according to a procedure described for CHO cells^76^ with the following minor modifications: After reaching ≈80% confluency, the cells were detached from the cell culture flask by trypsinization. After centrifugation (400*g*, 6 min), the cells were resuspended in L-15 medium with 1% BSA and the cell density was adjusted to 1.25 × 10^6^ (for the Nluc-Y_1_R constructs and Nluc-M_1_R(G176)) or 1.25 × 10^5^ (for Nluc-M_1_R(Nterm)) cells/mL. [^3^H]UR-MK299^25^ or [^3^H]NMS were used as radioligands for experiments at the Nluc-Y_1_R constructs or Nluc-M_1_R constructs, respectively. Nonspecific binding was assessed in the presence of either BIBO3304 (for the Nluc-Y_1_R constructs, 500-fold excess over the concentration of radioligand) or atropine (for the Nluc-M_1_R constructs, 1000-fold excess over the concentration of radioligand). The wells were prefilled with 20 μL of L-15 containing the respective radioligand (10-fold more concentrated than the final assay concentration) and 20 μL of L-15 (total binding) or 20 μL of L-15 containing the suitable competitive ligand (nonspecific binding). A 160 μL amount of the density-adjusted cell suspension was added to the wells, and the plate was incubated under gentle shaking at 23 °C for 90 min (for the Nluc-Y_1_R constructs) and 3 h (for the Nluc-M_1_R constructs), respectively.

Saturation binding experiments at Nluc-NTS_1_R(T227) and Nluc-AT_1_R(S186) were essentially conducted following a described protocol^44^ with modifications; in detail: saturation binding was investigated with [^3^H]UR-MK300 at intact HEK293T cells stably expressing Nluc-NTS_1_R(T227) and with [^3^H]UR-MK292 at intact HEK293T cells stably expressing Nluc-AT_1_R(S186). Experiments were performed at room temperature (rt) in white 96-well plates with clear bottoms (Corning Inc., Tewksbury, MA). Two days before the experiment, the plates were treated with poly-d-lysine hydrobromide (Sigma-Aldrich, Munich, Germany) for 10 min. Subsequently, the plates were washed once with PBS and left to dry overnight at rt. Dulbecco’s PBS (D-PBS) with Ca^2+^ and Mg^2+^ (1.8 mM CaCl_2_, 2.68 mM KCl, 1.47 mM KH_2_PO_4_, 3.98 mM MgSO_4_, 136.9 mM NaCl and 8.06 mM Na_2_HPO_4_), supplemented with 1% BSA and 100 μg/mL of the protease inhibitor bacitracin, served as binding buffer. Washing steps were performed using D-PBS at rt (prior to the incubation) or ice-cold (after incubation). Cells were seeded 1 day before the experiment in 100 μL of DMEM with 2 mM l-glutamine and 10% FCS at a density of 1 × 10^5^ cells/well. On the day of the experiment, the culture medium was carefully removed using a multichannel pipet (Transferpette S-12, Brand, Wertheim, Germany), and the cells were washed once with D-PBS (200 μL) and covered with binding buffer (160 μL). For the assessment of total binding, 20 μL of binding buffer and the same volume of binding buffer containing the radioligand (10-fold more concentrated than the final concentration) were added. Nonspecific binding was determined in the presence of NT(8–13) (for Nluc-NTS_1_R(T227)) or angiotensin II (for Nluc-AT_1_R(S186)) in 500-fold excess over the respective concentration of the radioligand by adding binding buffer (20 μL) containing the competitor (10-fold more concentrated than the final concentration) and binding buffer (20 μL) containing the radioligand (10-fold more concentrated than the final concentration). The plates were gently shaken for 2 h at rt. After incubation, the liquid was carefully removed using a multichannel pipet, the cells were washed twice with ice-cold D-PBS (200 μL) and treated with lysis solution (urea (8 M), acetic acid (3 M) and Triton X-100 (1%) in water) (25 μL). The plates were shaken for 20 min, a liquid scintillator (Ultima Gold (PerkinElmer, Waltham, MA)) (200 μL) was added, and the plates were sealed with a transparent sealing tape (permanent seal for microplates, PerkinElmer, prod. no. 1450-461). Complete mixing of scintillator and lysis solution was achieved by turning the plates upside down multiple times. The plates were kept in the dark for at least 30 min prior to the measurement of the radioactivity (dpm) with a MicroBeta2 plate counter (PerkinElmer, Rodgau, Germany). Radioligand competition binding experiments with [^3^H]UR-MK292 at intact CHO-AT1-Gα_16_-mtAEQ cells were performed as described.^44^

### BRET Binding Assay at Intact HEK293T Cells

BRET binding experiments were essentially performed as described^18^ with the following minor modifications: 1 day prior to the experiment, HEK293T cells stably expressing the respective Nluc-receptor fusion construct were detached from the cell culture dish by trypsinization, centrifuged (500*g*, 5 min), and resuspended in L-15 with 5% FCS and 10 mM HEPES (pH 7.4). After adjusting the cell density to 1.4 × 10^6^ cells/mL, the cells were seeded in a volume of 70 μL per well into white 96-well plates (Brand, Wertheim, Germany) and incubated overnight in a water-saturated atmosphere (no additional CO_2_). On the day of the experiment, serial dilutions of the fluorescent ligands and the competitors (all 10-fold more concentrated than the final assay concentration) were prepared in assay buffer consisting of L-15 medium with 10 mM HEPES (pH 7.4) and 2% BSA. For saturation binding experiments, 10 μL of assay buffer (total binding) or 10 μL of assay buffer containing a competitive ligand (for the assessment of nonspecific binding, for the Nluc-Y_1_R constructs: BIBO3304 in a 500-fold excess; for the Nluc-NTS_1_R constructs: SR142948 in a 100-fold excess; for the Nluc-AT_1_R constructs: candesartan in a 100-fold excess; for the Nluc-M_1_R constructs: atropine in a 500-fold excess; the excess always refers to the respective concentration of fluorescent ligand used) were added to the wells. Subsequently, 10 μL of assay buffer containing the investigated fluorescent ligand was added to all wells. After an incubation period of 60 min at 27 °C, 10 μL of the Nluc substrate furimazine (prediluted 1:1000 before use) was added and after an equilibration time of 5 min inside the plate reader (prewarmed to 27 °C), the measurement was started.

For competition binding experiments at Nluc-Y_1_R(Y192), Nluc-Y_1_R(Q291), Nluc-NTS_1_R(T227), and Nluc-M_1_R(G176), 10 μL of a solution of the investigated competitive ligand (various concentrations) and 10 μL of a solution of the suitable fluorescent ligand (for Nluc-Y_1_R(Y192)/ Nluc-Y_1_R(Q291): *c*_final_(**1**) = 0.5 nM; for Nluc-NTS_1_R(T227): *c*_final_(**2**) = 5 nM; for Nluc-M_1_R(G176): *c*_final_(**4**) = 5 nM) were added. For competition binding experiments at Nluc-AT_1_R(S186), cells were preincubated with the competitor for 30 min before the addition of the fluorescent ligand **3** (*c*_final_ = 10 nM). A positive control containing only fluorescent ligand and no competitor (100% value), as well as a negative control (buffer, 0% value), was included in every experiment. After an incubation period of 60 min at 27 °C, 10 μL of furimazine (prediluted 1:1000 before use) was added to the cells and after equilibration for 5 min, the measurement was started.

For kinetic saturation and competition binding experiments, 10 μL of the substrate furimazine (prediluted 1:1000 before use) and 10 μL of assay buffer (total binding) or 10 μL of a solution of the competitive ligand (nonspecific binding and competition binding experiments) were added at the same time. After a short equilibration (5 min), 10 μL of the solution of the investigated fluorescent ligand was added to the cells (final concentrations of the fluorescent ligands in competition binding experiments: see above) and the measurement was started immediately. For kinetic BRET competition binding experiments at Nluc-AT_1_R(S186), the cells were preincubated with the competitive ligand for 30 min as described above prior to the addition of the substrate and the fluorescent ligand **3**.

For recording association and dissociation kinetics, 20 μL of assay buffer (total binding) or 20 μL of assay buffer containing the competitive ligand (nonspecific binding, for Nluc-Y_1_R(Y192): BIBO3304, *c*_final_ = 500 nM; for Nluc-NTS_1_R(T227): SR142948, *c*_final_ = 2.5 μM; for the AT_1_R(S186): candesartan, *c*_final_ = 2.5 μM; for the M_1_R(G176): atropine, *c*_final_ = 5 μM) were added to the cells. After the addition of 10 μL of furimazine (prediluted 1:1000 before use), cells were equilibrated inside the thermostated plate reader (27 °C) for 5 min and the measurement was started. After the first cycle (*t* = 0 min), 50 μL of assay buffer containing the fluorescent ligands **1** (Nluc-Y_1_R(Y192), *c*_final_ = 0.5 nM), **2** (Nluc-NTS_1_R(T227), *c*_final_ = 10 nM), **3** (Nluc-AT_1_R(S186), *c*_final_ = 10 nM), or **4** (Nluc-M_1_R(G176), *c*_final_ = 5 nM) were added via the injector module. Dissociation was initiated at the indicated time points by the addition of 50 μL of assay buffer containing the competitive ligand (for Nluc-Y_1_R(Y192): BIBO3304, *c*_final_ = 500 nM; Nluc-NTS_1_R(T227): SR142948, *c*_final_ = 2.5 μM; Nluc-AT_1_R(S186): candesartan, *c*_final_ = 2.5 μM; Nluc-M_1_R(G176): atropine, *c*_final_ = 5 μM).

All BRET measurements were performed at a temperature of 27 °C using a TECAN GENiosPro or a TECAN InfiniteLumi plate reader (Tecan Austria GmbH, Grödig, Austria). The bioluminescence of the luciferase was detected using a 460 ± 25 nm band-pass (460/25 BP, GENiosPro) filter or a 460 ± 35 nm band-pass (460/35 BP, InfiniteLumi) filter. The emission originating from the fluorescent ligand was detected through a 610 nm long-pass (610 LP) filter with both readers. Integration times for equilibrium experiments were set to 100 ms for both channels except for experiments at Nluc-Y_1_R(Y192), where a longer integration time (300 ms) was used for both channels. Kinetic experiments (except for on–off-kinetics) were monitored using an integration time of 1000 ms for the 610 LP filter to reduce noise. For the determination of on–off-kinetics, the following integration times (460 BP/610 LP) were used: Nluc-Y_1_R(Y192) 1000 ms/1000 ms, Nluc-NTS_1_R(T227) and Nluc-AT_1_R(S186): 100 ms/500 ms, Nluc-M_1_R(G176): 1000 ms/1000 ms

#### Preparation of HEK293T Cell Homogenates

Cell homogenates of HEK293T cells stably expressing Nluc-AT_1_R(S186) or Nluc-NTS_1_R(T227) were prepared as described^77^ with the following minor modifications: after cell lysis and the centrifugation of the lysate (23 000 rpm, 4 °C, 45 min), the pellet was resuspended in ice-cold binding buffer (50 mM Tris-HCl, 1 mM EDTA, pH 7.4) and homogenized using a 1 mL syringe (Injekt-F, B. Braun Melsungen AG, Melsungen, Germany) and a needle with 0.4 mm diameter (BD Microlance, Becton Dickinson, Heidelberg, Germany). The protein concentration was determined by the Bradford method, and aliquots of the homogenates were stored at −80 °C until further use.

### BRET Binding Assay at HEK293T Cell Homogenates

To perform kinetic BRET saturation binding experiments at cell homogenates of HEK293T expressing Nluc-AT_1_R(S186) or Nluc-NTS_1_R(T227), the homogenate was thawed and centrifuged (16 000*g*, 4 °C, 10 min). The pellet was resuspended in ice-cold binding buffer, and the protein concentration was adjusted to 1.5 μg/μL (Nluc-AT_1_R(S186)) or 0.9 μg/μL (Nluc-NTS_1_R(T227)). Next, 10 μL of the concentration-adjusted cell homogenates was added to each well in a white 96-well plate (Brand, Wertheim, Germany) together with 60 μL of binding buffer. The assay was performed as described above following the protocol for BRET saturation binding experiments at intact cells using a Tecan GENiosPro plate reader. The integration times were set to 100 ms (460/25 BP filter) and 500 ms (610 LP filter).

### Fura-2 Ca^2+^ Assay

The Fura-2 calcium assays were essentially performed as previously described.^78^ Therefore, HEK293T cells stably expressing Nluc-NTS_1_R(T227) or Nluc-AT_1_R(S186) were incubated with Fura-2 AM (Merck Biochrom, Berlin, Germany). The Ca^2+^ responses were measured in cuvettes using a LS50B luminescence spectrophotometer (PerkinElmer, Rodgau, Germany).

### Data Analysis

Data analysis was performed using GraphPad Prism 8.0 (GraphPad Software Inc., San Diego, CA). Specific binding data (dpm) from radioligand saturation binding experiments were plotted against the free radioligand concentration and fitted by an equation for hyperbolic binding (“one site-specific binding”, GraphPad Prism 8.0) yielding *K*_d_ and *B*_max_ values. Total and nonspecific binding were analyzed simultaneously using the “one site-total and non-specific binding” model in Prism 8.0. The free radioligand concentration was calculated by subtraction of the amount of specifically bound radioligand from the total radioligand concentration. The number of binding sites per cell was calculated from the *B*_max_ values as described.^44^ Error propagation was calculated as described.^79^

Obtained data from radioligand competition binding experiments at the AT_1_ receptor were normalized to the radioactivity measured in the presence of the radioligand [^3^H]UR-MK292, but in the absence of competitor (100%) and analyzed by a four-parameter logistic equation (Prism 8.0) yielding pIC_50_ values. These were transformed into p*K*_i_ values using the Cheng–Prusoff equation (logarithmic form),^80^ for which the mean and SD was calculated.

For BRET experiments, the “raw BRET ratio” was calculated by dividing the emission detected through the 610 LP filter (acceptor) by the emission detected through the 460 BP filter (donor). A baseline correction was performed for all values by subtracting the BRET ratio of a buffer control, yielding “corrected BRET ratios”. For saturation binding experiments, total and nonspecific binding were fitted simultaneously applying a one-site binding model (“one site-total and non-specific binding”; Prism 8.0), which fits total binding by a hyperbolic curve and nonspecific binding by linear regression. Specific binding was fitted by an equation describing hyperbolic binding (“one site-specific binding”, Prism 8.0). The obtained *K*_d_ values were transformed into p*K*_d_ values, for which means and SEMs were calculated.

For competition binding experiments, the data were normalized to the BRET ratio obtained for the negative control (buffer, 0% value) and the BRET ratio obtained for wells containing fluorescent ligand, but no competitor (100% value). The normalized data were then fitted by a four-parameter logistic equation (Prism 8.0). The obtained pIC_50_ values were subsequently transformed into p*K*_i_ values by means of the Cheng–Prusoff equation (logarithmic form),^80^ for which means and SEMs were calculated.

Kinetic data were normalized to the BRET ratio before the addition of a fluorescent ligand (0%) and the maximal BRET ratio obtained after association reached a plateau (100%). The data from combined association and dissociation experiments were then analyzed by an “association then dissociation” fit (Prism 8.0) yielding estimates for *k*_on_, *k*_off_, and *K*_d_^kinetic^ values for each independent experiment. The obtained *K*_d_^kinetic^ values were transformed into p*K*_d_^kinetic^ values for every experiment, and means and SEMs were calculated for the p*K*_d_^kinetic^ values.

## Supporting Information Available

Binding isotherms from radioligand saturation binding experiments; structures of the competitive ligands used in the course of the study; snake plots of the investigated GPCRs with an indication of the insertion sites for Nluc; functional validation of Nluc-NTS_1_R(T227) and AT_1_R(S186) in a Fura-2 Ca^2+^ assay; displacement curve from radioligand competition binding experiments with **3** at the AT_1_R; BRET-based binding experiments with the muscarinic acetylcholine receptor ligands **6** and **7**; kinetic traces (BRET binding) of the binding of fluorescent ligands **1**–**4** to their respective targets; saturation binding experiments with **2** and **3** at cell homogenates of HEK293T cells expressing Nluc-NTS_1_R(T227) or Nluc-AT_1_R(S186); kinetic traces from BRET competition binding experiments at Nluc-NTS_1_R(T227) or Nluc-AT_1_R(S186); Supporting Tables: p*K*_d_ values of the used radioligands obtained from radioligand saturation binding experiments; p*K*_i_ values of reported standard antagonists from BRET competition binding experiments at Nluc-Y_1_R(Q291); p*K*_d_ values of the fluorescent muscarinic acetylcholine receptor ligands **6** and **7** from BRET saturation binding experiments; p*K*_d_ values of **2** and **3** from BRET saturation binding experiments at cell homogenates; synthesis and analytical characterization of fluorescent ligand **3** and BIBP3226 (PDF)

## References

[ref1] Fredriksson R.; Lagerström M. C.; Lundin L.-G.; Schiöth H. B. The G-protein-coupled receptors in the human genome form five main families. Phylogenetic analysis, paralogon groups, and fingerprints. Mol. Pharmacol. 2003, 63, 1256–1272. 10.1124/mol.63.6.1256. 12761335

[ref2] Venkatakrishnan A. J.; Deupi X.; Lebon G.; Tate C. G.; Schertler G. F.; Babu M. M. Molecular signatures of G-protein-coupled receptors. Nature 2013, 494, 185–194. 10.1038/nature11896. 23407534

[ref3] Peeters M. C.; van Westen G. J. P.; Li Q.; Ijzerman A. P. Importance of the extracellular loops in G protein-coupled receptors for ligand recognition and receptor activation. Trends Pharmacol. Sci. 2011, 32, 35–42. 10.1016/j.tips.2010.10.001. 21075459

[ref4] Santos R.; Ursu O.; Gaulton A.; Bento A. P.; Donadi R. S.; Bologa C. G.; Karlsson A.; Al-Lazikani B.; Hersey A.; Oprea T. I.; Overington J. P. A comprehensive map of molecular drug targets. Nat. Rev. Drug Discovery 2017, 16, 19–34. 10.1038/nrd.2016.230. 27910877 PMC6314433

[ref5] Hauser A. S.; Attwood M. M.; Rask-Andersen M.; Schiöth H. B.; Gloriam D. E. Trends in GPCR drug discovery: new agents, targets and indications. Nat. Rev. Drug Discovery 2017, 16, 829–842. 10.1038/nrd.2017.178. 29075003 PMC6882681

[ref6] Hoffmann C.; Castro M.; Rinken A.; Leurs R.; Hill S. J.; Vischer H. F. Ligand residence time at G-protein–coupled receptors—why we should take our time to study it. Mol. Pharmacol. 2015, 88, 552–560. 10.1124/mol.115.099671. 26152198

[ref7] Swinney D. C.; Haubrich B. A.; Van Liefde I.; Vauquelin G. The role of binding kinetics in GPCR drug discovery. Curr. Top. Med. Chem. 2015, 15, 2504–2522. 10.2174/1568026615666150701113054. 26126905

[ref8] Zhang R.; Monsma F. Binding kinetics and mechanism of action: toward the discovery and development of better and best in class drugs. Expert Opin. Drug Discovery 2010, 5, 1023–1029. 10.1517/17460441.2010.520700. 22827742

[ref9] Sridharan R.; Zuber J.; Connelly S. M.; Mathew E.; Dumont M. E. Fluorescent approaches for understanding interactions of ligands with G protein coupled receptors. Biochim. Biophys. Acta Biomembr. 2014, 1838, 15–33. 10.1016/j.bbamem.2013.09.005. PMC3926105 24055822

[ref10] Stoddart L. A.; White C. W.; Nguyen K.; Hill S. J.; Pfleger K. D. Fluorescence- and bioluminescence-based approaches to study GPCR ligand binding. Br. J. Pharmacol. 2016, 173, 3028–3037. 10.1111/bph.13316. 26317175 PMC5125978

[ref11] Zhang R.; Xie X. Tools for GPCR drug discovery. Acta Pharmacol. Sin. 2012, 33, 372–384. 10.1038/aps.2011.173. 22266728 PMC3312097

[ref12] Emami-Nemini A.; Roux T.; Leblay M.; Bourrier E.; Lamarque L.; Trinquet E.; Lohse M. J. Time-resolved fluorescence ligand binding for G protein–coupled receptors. Nat. Protoc. 2013, 8, 1307–1320. 10.1038/nprot.2013.073. 23764938

[ref13] Stoddart L. A.; Kilpatrick L. E.; Hill S. J. NanoBRET approaches to study ligand binding to GPCRs and RTKs. Trends Pharmacol. Sci. 2018, 39, 136–147. 10.1016/j.tips.2017.10.006. 29132917

[ref14] Tahtaoui C.; Parrot I.; Klotz P.; Guillier F.; Galzi J. L.; Hibert M.; Ilien B. Fluorescent pirenzepine derivatives as potential bitopic ligands of the human M1 muscarinic receptor. J. Med. Chem. 2004, 47, 4300–4315. 10.1021/jm040800a. 15294002

[ref15] Hall M. P.; Unch J.; Binkowski B. F.; Valley M. P.; Butler B. L.; Wood M. G.; Otto P.; Zimmerman K.; Vidugiris G.; Machleidt T.; Robers M. B.; Benink H. A.; Eggers C. T.; Slater M. R.; Meisenheimer P. L.; Klaubert D. H.; Fan F.; Encell L. P.; Wood K. V. Engineered luciferase reporter from a deep sea shrimp utilizing a novel imidazopyrazinone substrate. ACS Chem. Biol. 2012, 7, 1848–1857. 10.1021/cb3002478. 22894855 PMC3501149

[ref16] Stoddart L. A.; Johnstone E. K. M.; Wheal A. J.; Goulding J.; Robers M. B.; Machleidt T.; Wood K. V.; Hill S. J.; Pfleger K. D. G. Application of BRET to monitor ligand binding to GPCRs. Nat. Methods 2015, 12, 661–663. 10.1038/nmeth.3398. 26030448 PMC4488387

[ref17] Förster T. Zwischenmolekulare Energiewanderung und Fluoreszenz. Ann. Phys. 1948, 437, 55–75. 10.1002/andp.19484370105.

[ref18] Bartole E.; Grätz L.; Littmann T.; Wifling D.; Seibel U.; Buschauer A.; Bernhardt G. UR-DEBa242: a Py-5-labeled fluorescent multipurpose probe for investigations on the histamine H_3_ and H_4_ receptors. J. Med. Chem. 2020, 63, 5297–5311. 10.1021/acs.jmedchem.0c00160. 32420741

[ref19] Fernández-Dueñas V.; Qian M.; Argerich J.; Amaral C.; Risseeuw M. D. P.; Van Calenbergh S.; Ciruela F. Design, synthesis and characterization of a new series of fluorescent metabotropic glutamate receptor type 5 negative allosteric modulators. Molecules 2020, 25, 1532 10.3390/molecules25071532. PMC7180738 32230915

[ref20] Grätz L.; Tropmann K.; Bresinsky M.; Müller C.; Bernhardt G.; Pockes S. NanoBRET binding assay for histamine H_2_ receptor ligands using live recombinant HEK293T cells. Sci. Rep. 2020, 10, 13288 10.1038/s41598-020-70332-3. 32764682 PMC7414126

[ref21] Hoare B. L.; Bruell S.; Sethi A.; Gooley P. R.; Lew M. J.; Hossain M. A.; Inoue A.; Scott D. J.; Bathgate R. A. D. Multi-component mechanism of H2 relaxin binding to RXFP1 through NanoBRET kinetic analysis. iScience 2019, 11, 93–113. 10.1016/j.isci.2018.12.004. 30594862 PMC6309025

[ref22] Kozielewicz P.; Bowin C. F.; Turku A.; Schulte G. A NanoBRET-based binding assay for Smoothened allows real-time analysis of ligand binding and distinction of two binding sites for BODIPY-cyclopamine. Mol. Pharmacol. 2020, 97, 23–34. 10.1124/mol.119.118158. 31707356

[ref23] Zhao P.; Liang Y.-L.; Belousoff M. J.; Deganutti G.; Fletcher M. M.; Willard F. S.; Bell M. G.; Christe M. E.; Sloop K. W.; Inoue A.; Truong T. T.; Clydesdale L.; Furness S. G. B.; Christopoulos A.; Wang M.-W.; Miller L. J.; Reynolds C. A.; Danev R.; Sexton P. M.; Wootten D. Activation of the GLP-1 receptor by a non-peptidic agonist. Nature 2020, 577, 432–436. 10.1038/s41586-019-1902-z. 31915381

[ref24] Müller C.; Gleixner J.; Tahk M.-J.; Kopanchuk S.; Laasfeld T.; Weinhart M.; Schollmeyer D.; Betschart M. U.; Lüdeke S.; Koch P.; Rinken A.; Keller M. Structure-based design of high-affinity fluorescent probes for the neuropeptide Y Y_1_ receptor. J. Med. Chem. 2022, 65, 4832–4853. 10.1021/acs.jmedchem.1c02033. 35263541

[ref25] Keller M.; Weiss S.; Hutzler C.; Kuhn K. K.; Mollereau C.; Dukorn S.; Schindler L.; Bernhardt G.; König B.; Buschauer A. N^ω^-Carbamoylation of the argininamide moiety: an avenue to insurmountable NPY Y_1_ receptor antagonists and a radiolabeled selective high-affinity molecular tool ([^3^H]UR-MK299) with extended residence time. J. Med. Chem. 2015, 58, 8834–8849. 10.1021/acs.jmedchem.5b00925. 26466164

[ref26] Lindner D.; Walther C.; Tennemann A.; Beck-Sickinger A. G. Functional role of the extracellular N-terminal domain of neuropeptide Y subfamily receptors in membrane integration and agonist-stimulated internalization. Cell. Signal. 2009, 21, 61–68. 10.1016/j.cellsig.2008.09.007. 18845246

[ref27] Yang Z.; Han S.; Keller M.; Kaiser A.; Bender B. J.; Bosse M.; Burkert K.; Kögler L. M.; Wifling D.; Bernhardt G.; Plank N.; Littmann T.; Schmidt P.; Yi C.; Li B.; Ye S.; Zhang R.; Xu B.; Larhammar D.; Stevens R. C.; Huster D.; Meiler J.; Zhao Q.; Beck-Sickinger A. G.; Buschauer A.; Wu B. Structural basis of ligand binding modes at the neuropeptide Y Y_1_ receptor. Nature 2018, 556, 520–524. 10.1038/s41586-018-0046-x. 29670288 PMC5920736

[ref28] Pettersen E. F.; Goddard T. D.; Huang C. C.; Couch G. S.; Greenblatt D. M.; Meng E. C.; Ferrin T. E. UCSF Chimera-a visualization system for exploratory research and analysis. J. Comput. Chem. 2004, 25, 1605–1612. 10.1002/jcc.20084. 15264254

[ref29] White C. W.; Johnstone E. K. M.; See H. B.; Pfleger K. D. G. NanoBRET ligand binding at a GPCR under endogenous promotion facilitated by CRISPR/Cas9 genome editing. Cell. Signal. 2019, 54, 27–34. 10.1016/j.cellsig.2018.11.018. 30471466

[ref30] Keller M.; Erdmann D.; Pop N.; Pluym N.; Teng S.; Bernhardt G.; Buschauer A. Red-fluorescent argininamide-type NPY Y_1_ receptor antagonists as pharmacological tools. Bioorg. Med. Chem. 2011, 19, 2859–2878. 10.1016/j.bmc.2011.03.045. 21493077

[ref31] Liu M.; Richardson R. R.; Mountford S. J.; Zhang L.; Tempone M. H.; Herzog H.; Holliday N. D.; Thompson P. E. Identification of a cyanine-dye labeled peptidic ligand for Y_1_R and Y_4_R, based upon the neuropeptide Y C-terminal analogue, BVD-15. Bioconjugate Chem. 2016, 27, 2166–2175. 10.1021/acs.bioconjchem.6b00376. 27513006

[ref32] Richardson R. R.; Groenen M.; Liu M.; Mountford S. J.; Briddon S. J.; Holliday N. D.; Thompson P. E. Heterodimeric analogues of the potent Y1R antagonist 1229U91, lacking one of the pharmacophoric C-terminal structures, retain potent Y1R affinity and show improved selectivity over Y4R. J. Med. Chem. 2020, 63, 5274–5286. 10.1021/acs.jmedchem.0c00027. 32364733

[ref33] Keller M.; Bernhardt G.; Buschauer A. [^3^H]UR-MK136: a highly potent and selective radioligand for neuropeptide Y Y_1_ receptors. ChemMedChem. 2011, 6, 1566–1571. 10.1002/cmdc.201100197. 21732539

[ref34] Rudolf K.; Eberlein W.; Engel W.; Wieland H. A.; Willim K. D.; Entzeroth M.; Wienen W.; Beck-Sickinger A. G.; Doods H. N. The first highly potent and selective non-peptide neuropeptide Y Y_1_ receptor antagonist: BIBP3226. Eur. J. Pharmacol. 1994, 271, R11–R13. 10.1016/0014-2999(94)90822-2. 7705422

[ref35] Antal-Zimanyi I.; Bruce M. A.; Leboulluec K. L.; Iben L. G.; Mattson G. K.; McGovern R. T.; Hogan J. B.; Leahy C. L.; Flowers S. C.; Stanley J. A.; Ortiz A. A.; Poindexter G. S. Pharmacological characterization and appetite suppressive properties of BMS-193885, a novel and selective neuropeptide Y_1_ receptor antagonist. Eur. J. Pharmacol. 2008, 590, 224–232. 10.1016/j.ejphar.2008.06.032. 18573246

[ref36] Poindexter G. S.; Bruce M. A.; LeBoulluec K. L.; Monkovic I.; Martin S. W.; Parker E. M.; Iben L. G.; McGovern R. T.; Ortiz A. A.; Stanley J. A.; Mattson G. K.; Kozlowski M.; Arcuri M.; Antal-Zimanyi I. Dihydropyridine neuropeptide Y Y_1_ receptor antagonists. Bioorg. Med. Chem. Lett. 2002, 12, 379–382. 10.1016/S0960-894X(01)00761-2. 11814801

[ref37] Wielgosz-Collin G.; Duflos M.; Pinson P.; Le Baut G.; Renard P.; Bennejean C.; Boutin J.; Boulanger M. 8-Amino-5-nitro-6-phenoxyquinolines: potential non-peptidic neuropeptide Y receptor ligands. J. Enzyme Inhib. Med. Chem. 2002, 17, 449–453. 10.1080/1475636021000005758. 12683683

[ref38] Wright J.; Bolton G.; Creswell M.; Downing D.; Georgic L.; Heffner T.; Hodges J.; MacKenzie R.; Wise L. 8-Amino-6-(arylsulphonyl)-5-nitroquinolines: novel nonpeptide neuropeptide Y1 receptor antagonists. Biorg. Med. Chem. Lett. 1996, 6, 1809–1814. 10.1016/0960-894X(96)00319-8.

[ref39] Thal D. M.; Sun B.; Feng D.; Nawaratne V.; Leach K.; Felder C. C.; Bures M. G.; Evans D. A.; Weis W. I.; Bachhawat P.; Kobilka T. S.; Sexton P. M.; Kobilka B. K.; Christopoulos A. Crystal structures of the M1 and M4 muscarinic acetylcholine receptors. Nature 2016, 531, 335–340. 10.1038/nature17188. 26958838 PMC4915387

[ref40] White J. F.; Noinaj N.; Shibata Y.; Love J.; Kloss B.; Xu F.; Gvozdenovic-Jeremic J.; Shah P.; Shiloach J.; Tate C. G.; Grisshammer R. Structure of the agonist-bound neurotensin receptor. Nature 2012, 490, 508–513. 10.1038/nature11558. 23051748 PMC3482300

[ref41] Zhang H.; Unal H.; Gati C.; Han G. W.; Liu W.; Zatsepin N. A.; James D.; Wang D.; Nelson G.; Weierstall U.; Sawaya M. R.; Xu Q.; Messerschmidt M.; Williams G. J.; Boutet S.; Yefanov O. M.; White T. A.; Wang C.; Ishchenko A.; Tirupula K. C.; Desnoyer R.; Coe J.; Conrad C. E.; Fromme P.; Stevens R. C.; Katritch V.; Karnik S. S.; Cherezov V. Structure of the angiotensin receptor revealed by serial femtosecond crystallography. Cell 2015, 161, 833–844. 10.1016/j.cell.2015.04.011. 25913193 PMC4427029

[ref42] Keller M.; Mahuroof S. A.; Hong Yee V.; Carpenter J.; Schindler L.; Littmann T.; Pegoli A.; Hübner H.; Bernhardt G.; Gmeiner P.; Holliday N. D. Fluorescence labeling of neurotensin(8–13) via arginine residues gives molecular tools with high receptor affinity. ACS Med. Chem. Lett. 2020, 11, 16–22. 10.1021/acsmedchemlett.9b00462. 31938457 PMC6956362

[ref43] Gruber C. G.; Pegoli A.; Müller C.; Grätz L.; She X.; Keller M. Differently fluorescence-labelled dibenzodiazepinone-type muscarinic acetylcholine receptor ligands with high M_2_R affinity. RSC Med. Chem. 2020, 11, 823–832. 10.1039/D0MD00137F. 33479678 PMC7650007

[ref44] Keller M.; Kuhn K. K.; Einsiedel J.; Hübner H.; Biselli S.; Mollereau C.; Wifling D.; Svobodová J.; Bernhardt G.; Cabrele C.; Vanderheyden P. M. L.; Gmeiner P.; Buschauer A. Mimicking of arginine by functionalized *N*^ω^-carbamoylated arginine as a new broadly applicable approach to labeled bioactive peptides: high affinity angiotensin, neuropeptide Y, neuropeptide FF, and neurotensin receptor ligands as examples. J. Med. Chem. 2016, 59, 1925–1945. 10.1021/acs.jmedchem.5b01495. 26824643

[ref45] De Lean A.; Stadel J. M.; Lefkowitz R. J. A ternary complex model explains the agonist-specific binding properties of the adenylate cyclase-coupled β-adrenergic receptor. J. Biol. Chem. 1980, 255, 7108–7117. 10.1016/S0021-9258(20)79672-9. 6248546

[ref46] Bhuiyan M. A.; Ishiguro M.; Hossain M.; Nakamura T.; Ozaki M.; Miura S.; Nagatomo T. Binding sites of valsartan, candesartan and losartan with angiotensin II receptor 1 subtype by molecular modeling. Life Sci. 2009, 85, 136–140. 10.1016/j.lfs.2009.05.001. 19446572

[ref47] Le M. T.; Vanderheyden P. M.; Szaszák M.; Hunyady L.; Vauquelin G. Angiotensin IV is a potent agonist for constitutive active human AT_1_ receptors. Distinct roles of the N- and C-terminal residues of angiotensin II during AT_1_ receptor activation. J. Biol. Chem. 2002, 277, 23107–23110. 10.1074/jbc.C200201200. 12006574

[ref48] Verheijen I.; Fierens F. L.; Debacker J. P.; Vauquelin G.; Vanderheyden P. M. Interaction between the partially insurmountable antagonist valsartan and human recombinant angiotensin II type 1 receptors. Fundam. Clin. Pharmacol. 2000, 14, 577–585. 10.1111/j.1472-8206.2000.tb00443.x. 11206708

[ref49] Wingler L. M.; McMahon C.; Staus D. P.; Lefkowitz R. J.; Kruse A. C. Distinctive activation mechanism for angiotensin receptor revealed by a synthetic nanobody. Cell 2019, 176, 479–490. 10.1016/j.cell.2018.12.006. 30639100 PMC6367718

[ref50] Lang C.; Maschauer S.; Hübner H.; Gmeiner P.; Prante O. Synthesis and evaluation of a ^18^F-labeled diarylpyrazole glycoconjugate for the imaging of NTS1-positive tumors. J. Med. Chem. 2013, 56, 9361–9365. 10.1021/jm401491e. 24160350

[ref51] Lundquist; Dix T. A. Synthesis and human neurotensin receptor binding activities of neurotensin(8–13) analogues containing position 8 α-azido-*N*-alkylated derivatives of ornithine, lysine, and homolysine. J. Med. Chem. 1999, 42, 4914–4918. 10.1021/jm9903444. 10579853

[ref52] Schindler L.; Bernhardt G.; Keller M. Modifications at Arg and Ile give neurotensin(8–13) derivatives with high stability and retained NTS_1_ receptor affinity. ACS Med. Chem. Lett. 2019, 10, 960–965. 10.1021/acsmedchemlett.9b00122. 31223455 PMC6580558

[ref53] Gully D.; Labeeuw B.; Boigegrain R.; Oury-Donat F.; Bachy A.; Poncelet M.; Steinberg R.; Suaud-Chagny M. F.; Santucci V.; Vita N.; Pecceu F.; Labbé-Jullié C.; Kitabgi P.; Soubrié P.; Le Fur G.; Maffrand J. P. Biochemical and pharmacological activities of SR 142948A, a new potent neurotensin receptor antagonist. J. Pharmacol. Exp. Ther. 1997, 802–812. 9023294

[ref54] Abdul-Ridha A.; López L.; Keov P.; Thal D. M.; Mistry S. N.; Sexton P. M.; Lane J. R.; Canals M.; Christopoulos A. Molecular determinants of allosteric modulation at the M_1_ muscarinic acetylcholine receptor. J. Biol. Chem. 2014, 289, 6067–6079. 10.1074/jbc.M113.539080. 24443568 PMC3937673

[ref55] Dong G. Z.; Kameyama K.; Rinken A.; Haga T. Ligand binding properties of muscarinic acetylcholine receptor subtypes (m1-m5) expressed in baculovirus-infected insect cells. J. Pharmacol. Exp. Ther. 1995, 378–384. 7616422

[ref56] Fish I.; Stößel A.; Eitel K.; Valant C.; Albold S.; Huebner H.; Möller D.; Clark M. J.; Sunahara R. K.; Christopoulos A.; Shoichet B. K.; Gmeiner P. Structure-based design and discovery of new M_2_ receptor agonists. J. Med. Chem. 2017, 60, 9239–9250. 10.1021/acs.jmedchem.7b01113. 29094937 PMC5836741

[ref57] Jakubík J.; Bacáková L.; El-Fakahany E. E.; Tucek S. Positive cooperativity of acetylcholine and other agonists with allosteric ligands on muscarinic acetylcholine receptors. Mol. Pharmacol. 1997, 52, 172–179. 10.1124/mol.52.1.172. 9224827

[ref58] Matucci R.; Bellucci C.; Martino M. V.; Nesi M.; Manetti D.; Welzel J.; Bartz U.; Holze J.; Tränkle C.; Mohr K.; Mazzolari A.; Vistoli G.; Dei S.; Teodori E.; Romanelli M. N. Carbachol dimers with primary carbamate groups as homobivalent modulators of muscarinic receptors. Eur. J. Pharmacol. 2020, 883, 173183 10.1016/j.ejphar.2020.173183. 32534072

[ref59] Richards M. H.; van Giersbergen P. L. Human muscarinic receptors expressed in A9L and CHO cells: activation by full and partial agonists. Br. J. Pharmacol. 1995, 114, 1241–1249. 10.1111/j.1476-5381.1995.tb13339.x. 7620715 PMC1510344

[ref60] Buckley N. J.; Bonner T. I.; Buckley C. M.; Brann M. R. Antagonist binding properties of five cloned muscarinic receptors expressed in CHO-K1 cells. Mol. Pharmacol. 1989, 469–476. 2704370

[ref61] Christopoulos A.; Pierce T. L.; Sorman J. L.; El-Fakahany E. E. On the unique binding and activating properties of xanomeline at the M_1_ muscarinic acetylcholine receptor. Mol. Pharmacol. 1998, 1120–1130. 9614217

[ref62] Fruchart-Gaillard C.; Mourier G.; Marquer C.; Ménez A.; Servent D. Identification of various allosteric interaction sites on M_1_ muscarinic receptor using ^125^I-Met35-oxidized muscarinic toxin 7. Mol. Pharmacol. 2006, 69, 1641–1651. 10.1124/mol.105.020883. 16439611

[ref63] Huang F.; Buchwald P.; Browne C. E.; Farag H. H.; Wu W. M.; Ji F.; Hochhaus G.; Bodor N. Receptor binding studies of soft anticholinergic agents. AAPS PharmSci. 2001, 3, 44–56. 10.1208/ps030430. PMC2751219 12049493

[ref64] Keller M.; Tränkle C.; She X.; Pegoli A.; Bernhardt G.; Buschauer A.; Read R. W. M_2_ Subtype preferring dibenzodiazepinone-type muscarinic receptor ligands: effect of chemical homo-dimerization on orthosteric (and allosteric?) binding. Bioorg. Med. Chem. 2015, 23, 3970–3990. 10.1016/j.bmc.2015.01.015. 25650309

[ref65] Keov P.; López L.; Devine S. M.; Valant C.; Lane J. R.; Scammells P. J.; Sexton P. M.; Christopoulos A. Molecular mechanisms of bitopic ligand engagement with the M_1_ muscarinic acetylcholine receptor. J. Biol. Chem. 2014, 289, 23817–23837. 10.1074/jbc.M114.582874. 25006252 PMC4156061

[ref66] Del Bello F.; Barocelli E.; Bertoni S.; Bonifazi A.; Camalli M.; Campi G.; Giannella M.; Matucci R.; Nesi M.; Pigini M.; Quaglia W.; Piergentili A. 1,4-Dioxane, a suitable scaffold for the development of novel M_3_ muscarinic receptor antagonists. J. Med. Chem. 2012, 55, 1783–1787. 10.1021/jm2013216. 22243489

[ref67] Esqueda E. E.; Gerstin E. H. Jr.; Griffin M. T.; Ehlert F. J. Stimulation of cyclic AMP accumulation and phosphoinositide hydrolysis by M_3_ muscarinic receptors in the rat peripheral lung. Biochem. Pharmacol. 1996, 52, 643–658. 10.1016/0006-2952(96)00339-5. 8759038

[ref68] Kilpatrick L. E.; Friedman-Ohana R.; Alcobia D. C.; Riching K.; Peach C. J.; Wheal A. J.; Briddon S. J.; Robers M. B.; Zimmerman K.; Machleidt T.; Wood K. V.; Woolard J.; Hill S. J. Real-time analysis of the binding of fluorescent VEGF_165_a to VEGFR2 in living cells: effect of receptor tyrosine kinase inhibitors and fate of internalized agonist-receptor complexes. Biochem. Pharmacol. 2017, 136, 62–75. 10.1016/j.bcp.2017.04.006. 28392095 PMC5457915

[ref69] Peach C. J.; Kilpatrick L. E.; Woolard J.; Hill S. J. Comparison of the ligand-binding properties of fluorescent VEGF-A isoforms to VEGF receptor 2 in living cells and membrane preparations using NanoBRET. Br. J. Pharmacol. 2019, 176, 3220–3235. 10.1111/bph.14755. 31162634 PMC6692582

[ref70] Hein L.; Meinel L.; Pratt R. E.; Dzau V. J.; Kobilka B. K. Intracellular trafficking of angiotensin II and its AT_1_ and AT_2_ receptors: evidence for selective sorting of receptor and ligand. Mol. Endocrinol. 1997, 11, 1266–1277. 10.1210/mend.11.9.9975. 9259318

[ref71] Vandenbulcke F.; Nouel D.; Vincent J. P.; Mazella J.; Beaudet A. Ligand-induced internalization of neurotensin in transfected COS-7 cells: differential intracellular trafficking of ligand and receptor. J. Cell Sci. 2000, 113, 2963–2975. 10.1242/jcs.113.17.2963. 10934036

[ref72] Park P. S. H.; Lodowski D. T.; Palczewski K. Activation of G protein-coupled receptors: beyond two-state models and tertiary conformational changes. Annu. Rev. Pharmacol. Toxicol. 2008, 48, 107–141. 10.1146/annurev.pharmtox.48.113006.094630. 17848137 PMC2639654

[ref73] Samama P.; Cotecchia S.; Costa T.; Lefkowitz R. J. A mutation-induced activated state of the β_2_-adrenergic receptor. Extending the ternary complex model. J. Biol. Chem. 1993, 268, 4625–4636. 10.1016/S0021-9258(18)53442-6. 8095262

[ref74] Tang T.; Tan Q.; Han S.; Diemar A.; Löbner K.; Wang H.; Schüss C.; Behr V.; Mörl K.; Wang M.; Chu X.; Yi C.; Keller M.; Kofoed J.; Reedtz-Runge S.; Kaiser A.; Beck-Sickinger A. G.; Zhao Q.; Wu B. Receptor-specific recognition of NPY peptides revealed by structures of NPY receptors. Sci. Adv. 2022, 8, eabm1232 10.1126/sciadv.abm1232. 35507650 PMC9067930

[ref75] Kozielewicz P.; Turku A.; Bowin C.-F.; Petersen J.; Valnohova J.; Cañizal M. C. A.; Ono Y.; Inoue A.; Hoffmann C.; Schulte G. Structural insight into small molecule action on Frizzleds. Nat. Commun. 2020, 11, 414 10.1038/s41467-019-14149-3. 31964872 PMC6972889

[ref76] Pegoli A.; She X.; Wifling D.; Hubner H.; Bernhardt G.; Gmeiner P.; Keller M. Radiolabeled dibenzodiazepinone-type antagonists give evidence of dualsteric binding at the M_2_ muscarinic acetylcholine receptor. J. Med. Chem. 2017, 60, 3314–3334. 10.1021/acs.jmedchem.6b01892. 28388054

[ref77] Bartole E.; Littmann T.; Tanaka M.; Ozawa T.; Buschauer A.; Bernhardt G. [^3^H]UR-DEBa176: a 2,4-diaminopyrimidine-type radioligand enabling binding studies at the human, mouse, and rat histamine H_4_ Receptors. J. Med. Chem. 2019, 62, 8338–8356. 10.1021/acs.jmedchem.9b01342. 31469288

[ref78] Müller M.; Knieps S.; Geßele K.; Dove S.; Bernhardt G.; Buschauer A. Synthesis and neuropeptide Y Y_1_ receptor antagonistic activity of N,N-disubstituted ω-guanidino- and ω-aminoalkanoic acid amides. Arch. Pharm. 1997, 330, 333–342. 10.1002/ardp.19973301104. 9431025

[ref79] Grätz L.; Laasfeld T.; Allikalt A.; Gruber C. G.; Pegoli A.; Tahk M.-J.; Tsernant M.-L.; Keller M.; Rinken A. BRET- and fluorescence anisotropy-based assays for real-time monitoring of ligand binding to M2 muscarinic acetylcholine receptors. Biochim. Biophys. Acta Mol. Cell Res. 2021, 1868, 118930 10.1016/j.bbamcr.2020.118930. 33347921

[ref80] Cheng Y.-C.; Prusoff W. H. Relationship between the inhibition constant (*K*_I_) and the concentration of inhibitor which causes 50% inhibition (*I*_50_) of an enzymatic reaction. Biochem. Pharmacol. 1973, 22, 3099–3108. 10.1016/0006-2952(73)90196-2. 4202581

