# Supplementary material for: Insertion of Nanoluc into the Extracellular Loops as a Complementary Method To Establish BRET-Based Binding Assays for GPCRs
Source: ACS Pharmacol Transl Sci. 2022 Oct 31;5(11):1142–55. doi: 10.1021/acsptsci.2c00162 (PMC9667534; doi:10.1021/acsptsci.2c00162)
Supplement: Supplementary file 1 — pt2c00162_si_001.pdf [file pt2c00162_si_001.pdf]

Supporting Information

Insertion of Nanoluc into the extracellular loops as a complementary method to establish BRET-based binding assays for GPCRs

Lukas Grätz[a,1,*], *Christoph Müller*[a], *Andrea Pegoli*[a,2], *Lisa Schindler*[a], *Günther Bernhardt*[a], *Timo Littmann*[a,3]

[a] Institute of Pharmacy, Faculty of Chemistry and Pharmacy, University of Regensburg, Universitätsstrasse 31, D-93053 Regensburg, Germany

[1] Author's present address: Section of Receptor Biology & Signaling, Department of Physiology & Pharmacology, Karolinska Institutet, S-17165, Stockholm, Sweden

[2] Author's present address: Ramboll Italy Srl, Viale Edoardo Jenner 53, 20159 Milano, Italy

[3] Author's present address: AbbVie Deutschland GmbH & Co. KG, 67061 Ludwigshafen am Rhein, Germany

*: corresponding author

E-Mail address:

Lukas Grätz: lukas.graetz@ki.se

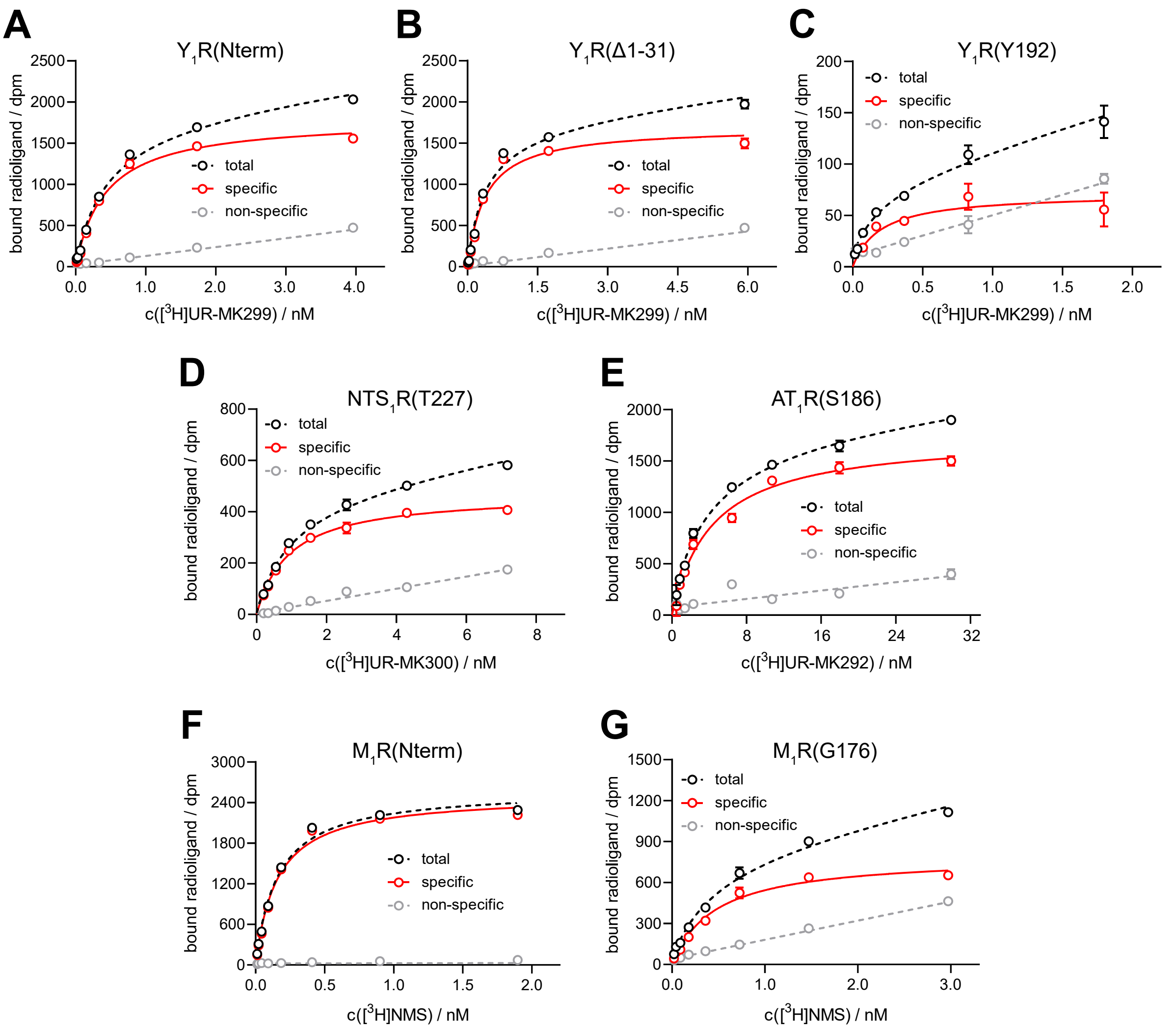

Supporting Figure S1: Binding isotherms from radioligand saturation binding experiments at intact HEK293T cells stably expressing Nluc-Y_1R(Nterm) (**A**), Nluc-Y_1R(Δ1-31) (**B**), Nluc-Y_1R(Y192) (**C**), Nluc-NTS_1R(T227) (**D**), Nluc-AT_1R(S186) (**E**), Nluc-M_1R(Nterm) (**F**) or Nluc-M_1R(G176) (**G**). [^{3}H]UR-MK299[1] (**A**-**C**), [^{3}H]UR-MK300[2] (**D**), [^{3}H]UR-MK292[2] (**E**) and [^{3}H]NMS (**F**, **G**) were used as radioligands. Non-specific binding was measured in the presence of an excess of BIBO3304 (500-fold; **A**-**C**), NT(8-13) (500-fold; **D**), angiotensin II (500-fold, **E**) or atropine (1000-fold, **F**, **G**). Presented data are shown as means ± errors from one representative of three independent experiments, each performed in triplicate. Error bars of total and non-specific binding represent the SEM, whereas error bars of specific binding represent propagated errors.

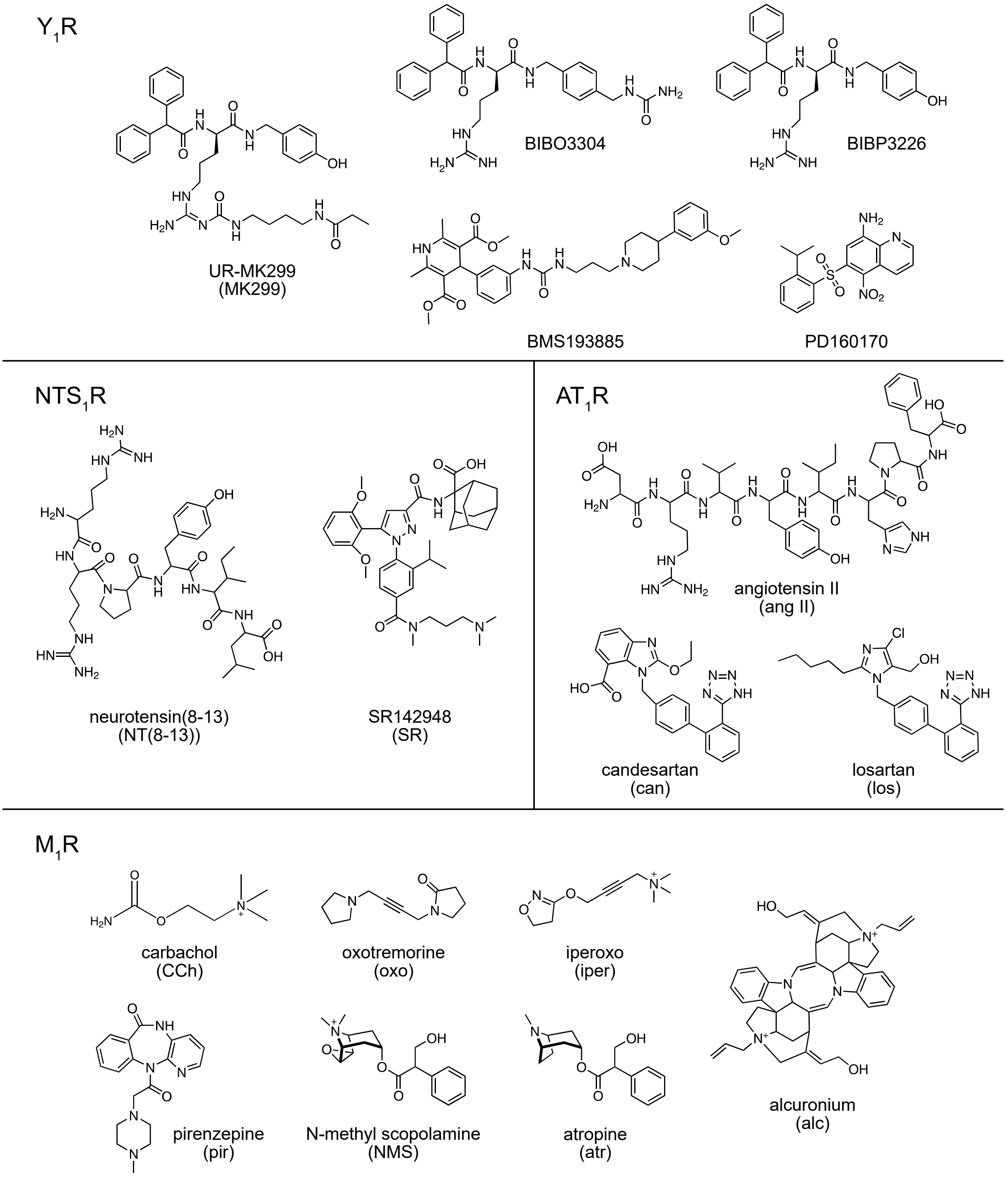

Supporting Figure S2: Structures of the competitive ligands used in this study.

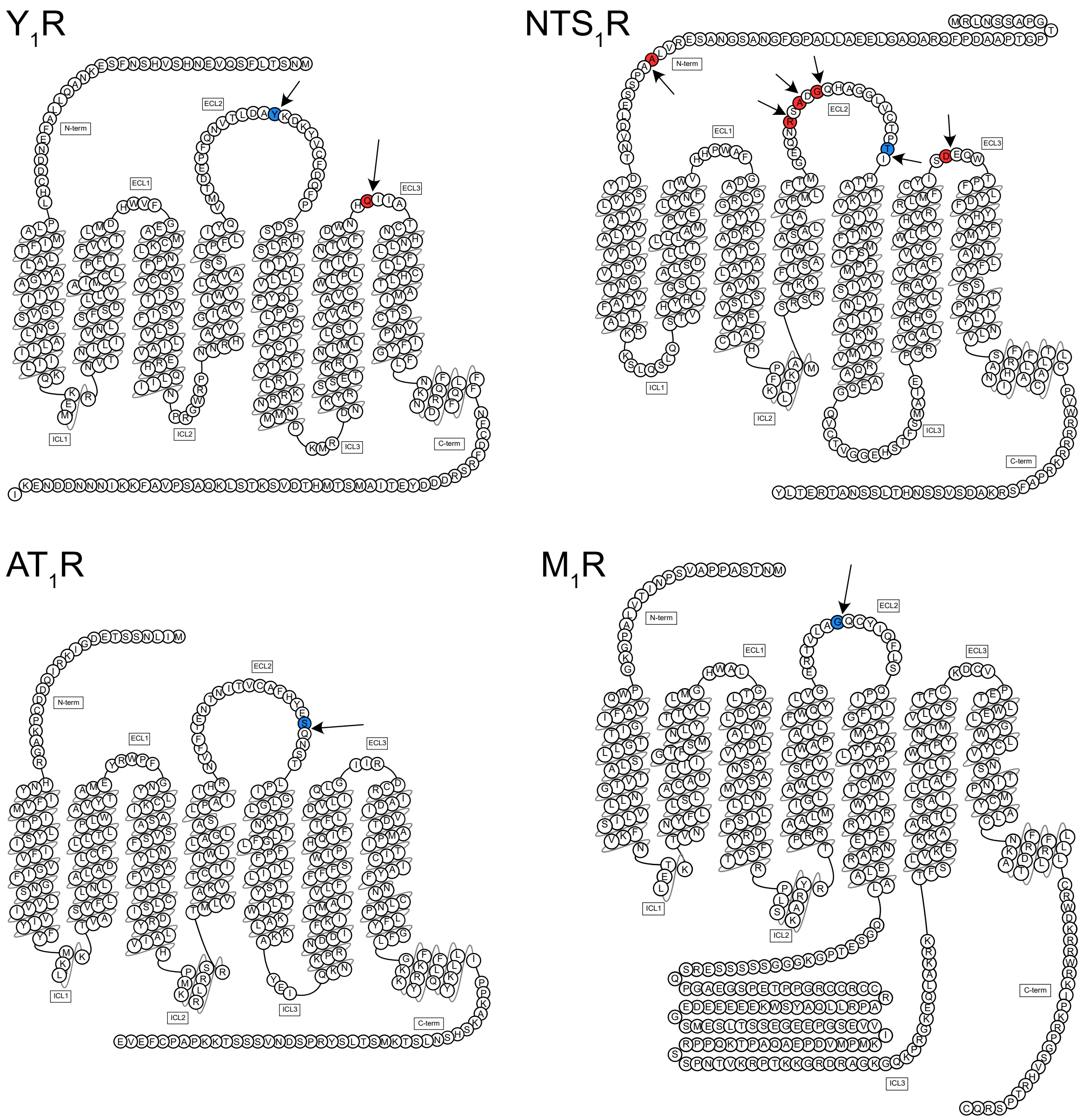

Supporting Figure S3: Snake plots of the Y_1R, NTS_1R, AT_1R and M_1R.[3-4] Insertion of Nluc after the residues in blue yielded the fusion proteins, which were investigated in this study in detail (Nluc-Y_1R(Y192), Nluc-NTS_1R(T227), Nluc-AT_1R(S186), Nluc-M_1R(G176)). Constructs obtained by insertion of the luciferase after the red coloured residues were omitted after an initial screening.

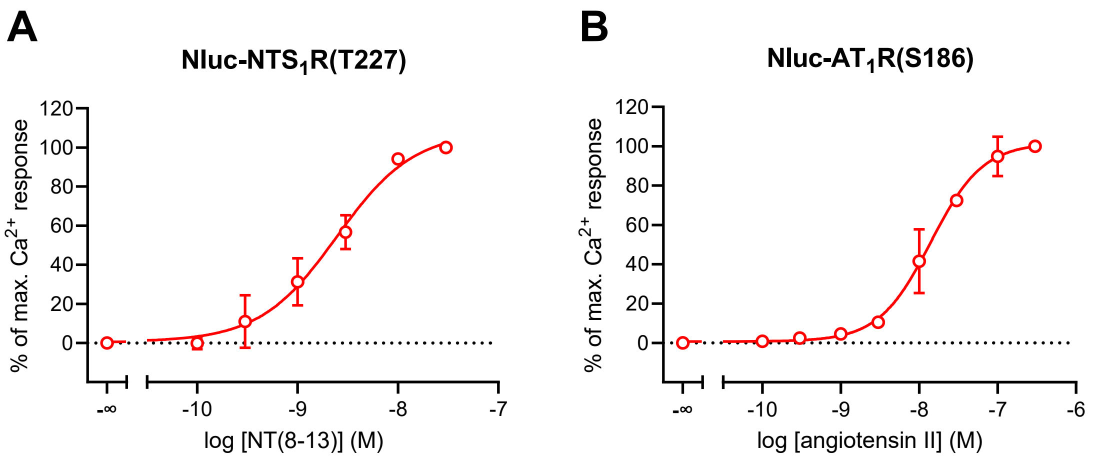

Supporting Figure S4: Functional validation of Nluc-NTS$_1$R(T227) (**A**) and Nluc-AT$_1$R(S186) (**B**) in a Fura-2 Ca^{2+} assay using NT(8-13) (**A**) or angiotensin II (**B**) as reference agonists. Experiments were conducted in HEK293T cells, stably expressing the indicated construct. Data are presented as means ± SD of two individual experiments (performed in singlet).

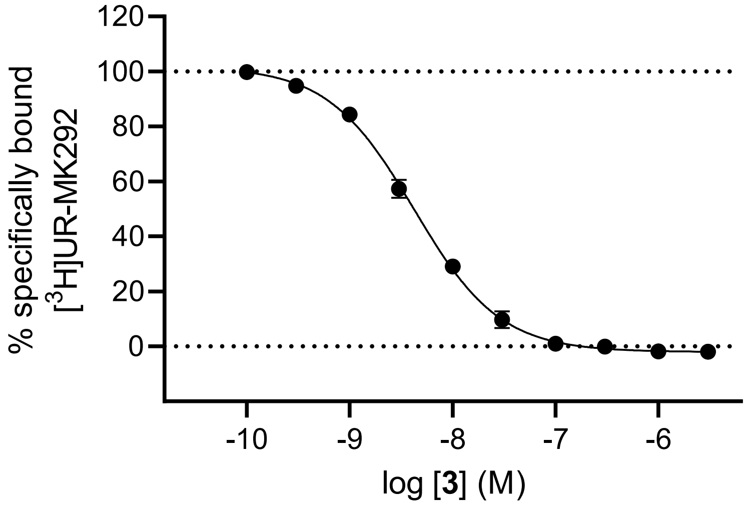

Supporting Figure S5: Displacement curve from radioligand competition binding experiments with radioligand [^{3}H]UR-MK292[2] (c = 1 nM) and the fluorescent ligand **3**, performed in intact CHO-AT$_1$-Gα_{16}-mtAEQ cells. Data are shown as means ± SD of two individual experiments, each performed in triplicate.

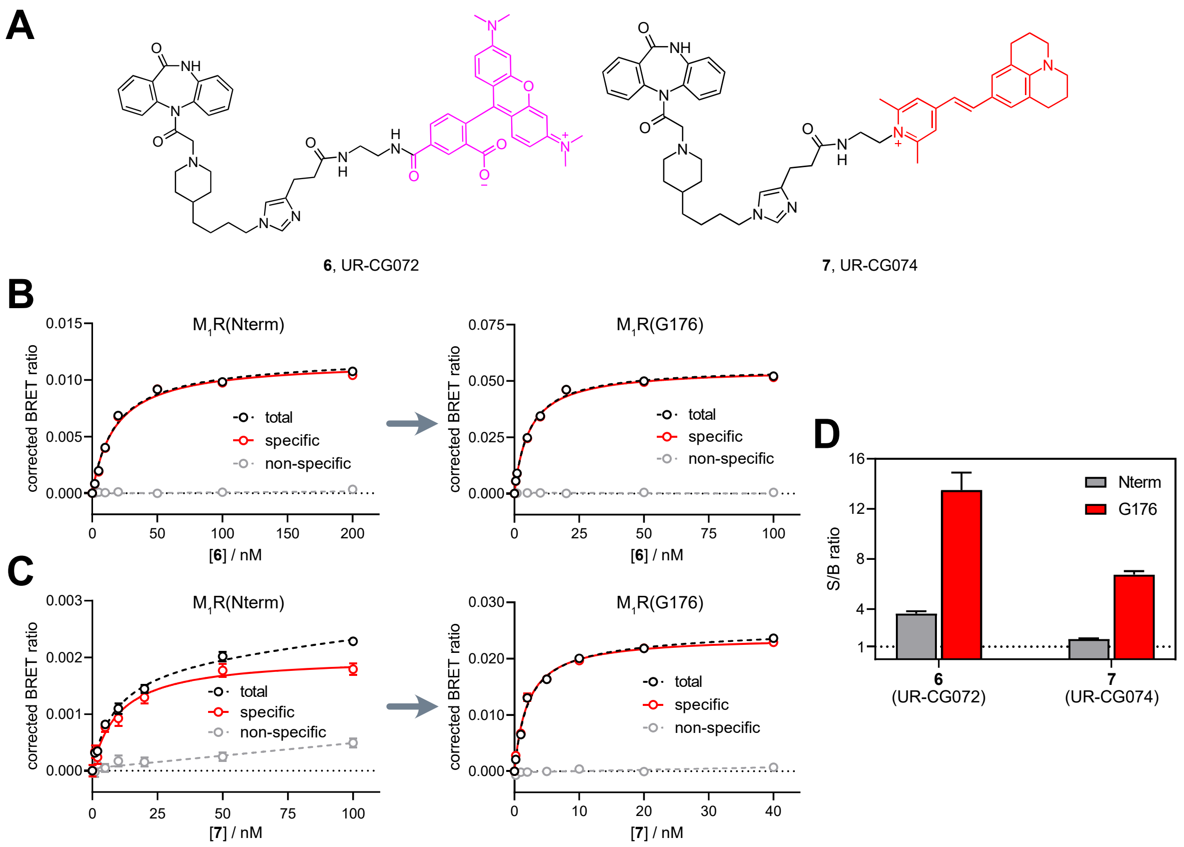

Supporting Figure S6: Characterization of the fluorescent MR ligands **6** and **7** in BRET binding assays at the M_1R. (**A**) Structures of the previously reported TAMRA-labeled MR ligand **6** (UR-CG072) and the Py-1-labeled MR ligand **7** (UR-CG074)[5];(**B**, **C**) Saturation isotherms from BRET binding experiments with **6** and **7** at HEK293T cells stably expressing Nluc-M_1R(Nterm) or Nluc-M_1R(G176). Non-specific binding was measured in the presence of a 500-fold excess of atropine (over the concentration of fluorescent ligand). Data represent means ± errors from one representative experiment of three independent experiments (performed in triplicate). Error bars of total and non-specific binding represent the SEM, whereas error bars of specific binding represent propagated errors. Corresponding pK_d values are listed in **Supporting Table S3**; (**D**) S/B ratios from BRET saturation binding experiments with **6** and **7** at HEK293T cells stably expressing Nluc-M_1R(Nterm) or Nluc-M_1R(G176). Data represent means ± SEM of three independent experiments, each performed in triplicate.

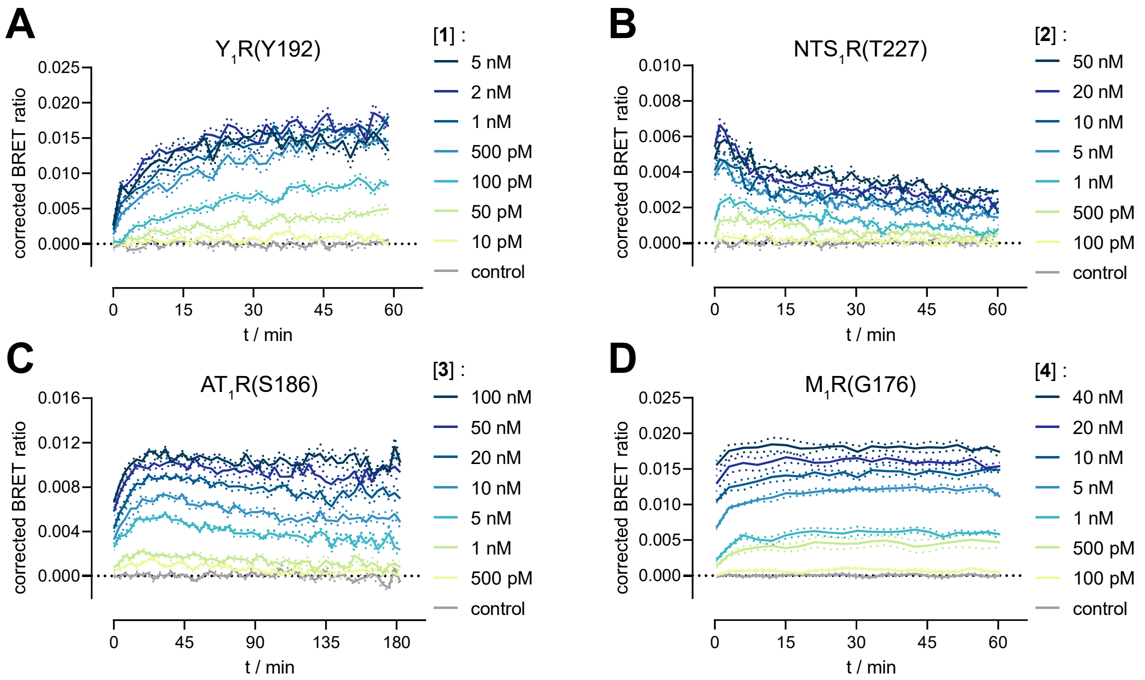

Supporting Figure S7: Kinetic traces of the binding of the fluorescent ligands **1**, **2**, **3** or **4** to Nluc-Y_1R(Y192) (**A**), Nluc-NTS_1R(T227) (**B**), Nluc-AT_1R(S186) (**C**) or Nluc-M_1R(G176) (**D**) respectively, stably expressed in intact HEK293T cells. The fluorescent ligands **1**-**4** were added at the timepoint t = 0 min. Data shown represent means ± propagated errors from kinetic saturation binding experiments. Presented data are representative of at least three independent experiments, each performed in triplicate.

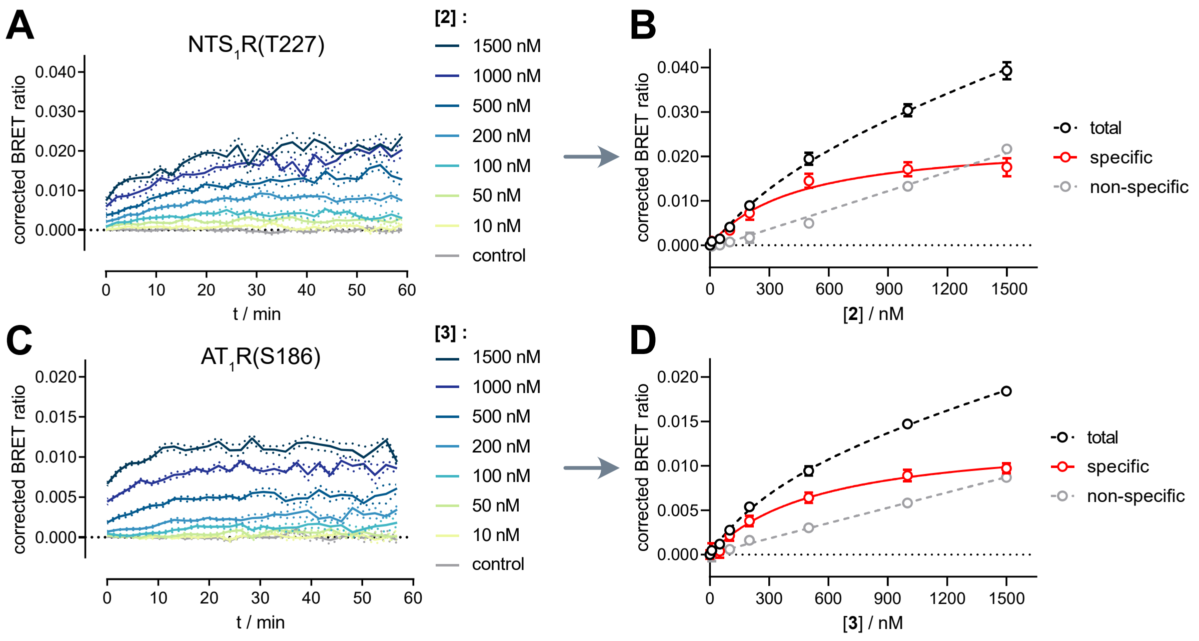

Supporting Figure S8: Saturation binding experiments with **2** or **3** at cell homogenates of HEK293T cells stably expressing Nluc-NTS$_1$R(T227) (**A, B**) or Nluc-AT$_1$R(S186) (**C, D**). (**A**, **C**) Kinetic traces of the specific binding of **2** to Nluc-NTS$_1$R(T227) (**A**) or **3** to Nluc-AT$_1$R(S186) (**C**) in HEK293T cell homogenates; the fluorescent ligands **2** or **3** were added at the timepoint t = 0 min; (**B**, **D**) Saturation isotherms of **2** (**B**) and **3** (**D**) corresponding to the kinetic traces in **A** and **C**. Shown data represent values after the last measured time point. Corresponding pK_d values are listed in **Supporting Table S4**. Data are presented as means ± errors and are representative of two independent experiments performed in triplicate. Errors of total and non-specific binding in **B** and **D** represent the SEM. Errors of specific binding in **A**-**D** represent propagated errors.

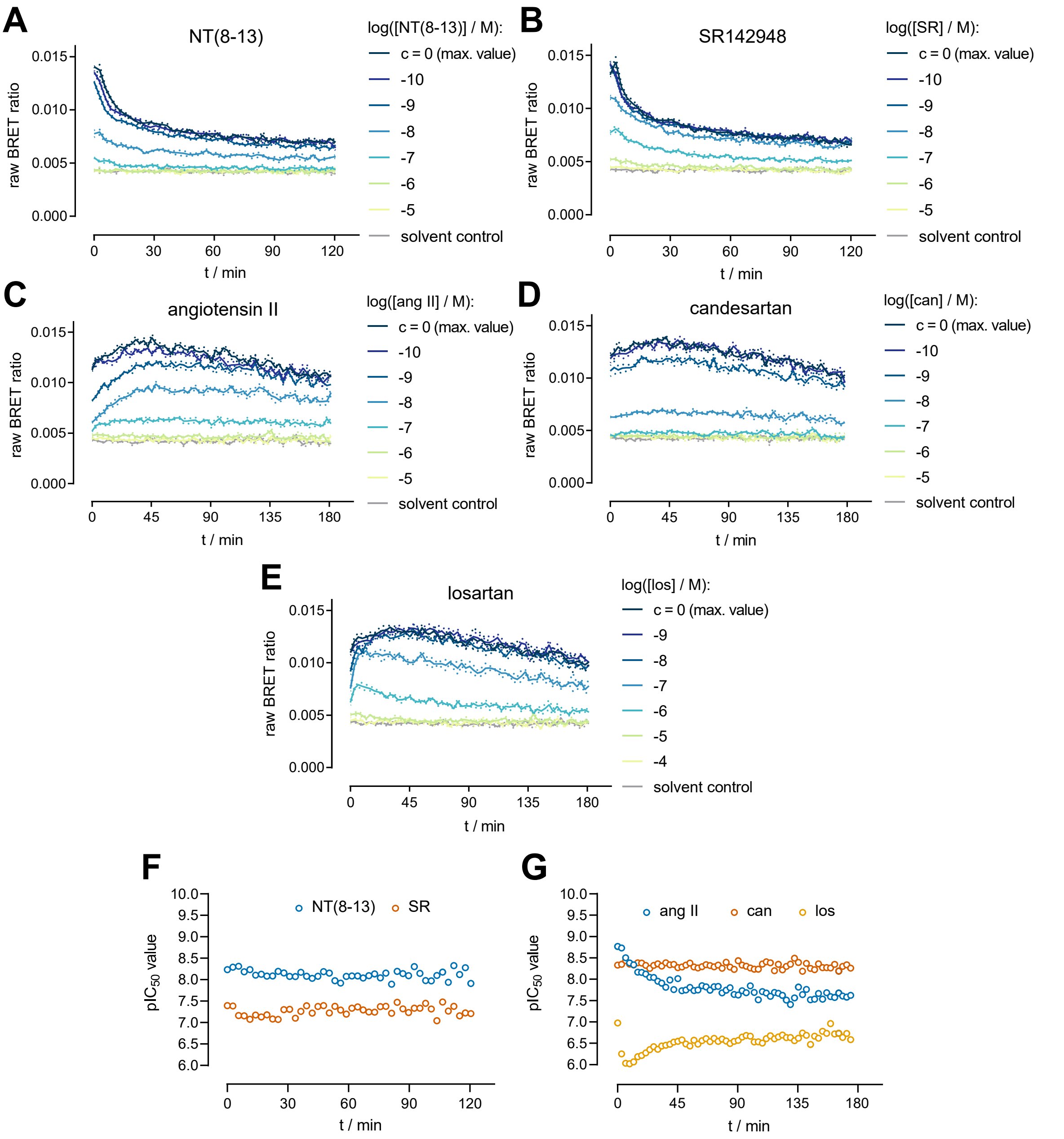

Supporting Figure S9: Kinetic traces from BRET competition binding experiments at Nluc-NTS$_1$R(T227) (**A**-**B**) or Nluc-AT$_1$R(S186) (**C**-**E**). Graphs **A** and **B** show exemplary kinetic competition binding experiments with NT(8-13) (**A**) or SR142948 (**B**) and the fluorescent ligand **2** (c = 5 nM) at Nluc-NTS$_1$R(T227), stably expressed in HEK293T cells. Graphs **C**-**E** show exemplary kinetic competition binding experiments with angiotensin II (**C**), candesartan (**D**) or losartan (**E**) and the fluorescent ligand **3** (c = 10 nM) at Nluc-AT$_1$R(S186), stably expressed in HEK293T cells. A negative control (solvent control) and a maximal value control (containing the fluorescent ligand **2** (**A**, **B**) or **3** (**C**-**E**) (c(**2**) = 5 nM; c(**3**) = 10 nM) but no competitor) were included in every experiment. The fluorescent ligands **2** (for **A** and **B**) or **3** (**C**-**E**) were added at the timepoint t = 0 min. Data are presented as means ± SEM and are representative of at least two independent experiments performed in triplicate; (**F**, **G**) Time-dependent change of the pIC$_{50}$ value of the investigated NTS$_1$R (**F**) and AT$_1$R (**G**) ligands. Shown data correspond to the kinetic competition binding experiments shown in **A**-**E**.

Supporting Table S1: pK_d values of the radioligands [^{3}H]UR-MK299, [^{3}H]UR-MK300, [^{3}H]UR-MK292 and [^{3}H]NMS obtained from radioligand saturation binding experiments.

receptor	radioligand	receptor (construct)	pK_d	N
Y_1R	[^{3}H]UR-MK299	Y_1R(wild-type)	9.85, 10.36[a]	---
		Nluc-Y_1R(Nterm)	9.28 ± 0.04[b]	3
		Nluc-Y_1R(Δ1-31)	9.45 ± 0.01[b]	3
		Nluc-Y_1R(Y192)	9.78 ± 0.08[b]	3
NTS_1R	[^{3}H]UR-MK300	NTS_1R(wild-type)	9.24, 9.29[c]	---
		Nluc-NTS_1R(T227)	8.81 ± 0.11[b]	3
AT_1R	[^{3}H]UR-MK292	AT_1R(wild-type)	9.03[c]	---
		Nluc-AT_1R(S186)	8.30 ± 0.08[b]	3
M_1R	[^{3}H]NMS	M_1R(wild-type)	9.77[d]	---
		Nluc-M_1R(Nterm)	9.80 ± 0.01[b]	3
		Nluc-M_1R(G176)	9.37 ± 0.13[b]	3

[a,c,d]Indicated pK_d values represent data from literature determined by radioligand saturation binding experiments at unmodified receptors; [a]Keller et *al.*[1]; [b]determined by radioligand saturation binding experiments with the indicated radioligand at intact HEK293T cells expressing the respective construct. Data are shown as means ± SEM of N independent experiments, performed in triplicate; [c]Keller et *al.*[2]; [d]Gruber et *al.*[5]

Supporting Table S2: pK_i values of reported Y_1R ligands from BRET competition binding experiments with **1** (c = 0.5 nM) at Nluc-Y_1R(Q291).

compound	pK_i (BRET, Nluc-Y_1R(Q291))[a]	*N*
UR-MK299	10.06 ± 0.07	3
BIBO3304	8.67 ± 0.23	3
BIBP3226	7.99 ± 0.10	3

[a]Determined by BRET competition binding experiments at Nluc-Y_1R(Q291) stably expressed in HEK293T cells using **1** as the fluorescent ligand (c = 0.5 nM, K_d = 0.29 nM). Data represent means ± SEM of *N* independent experiments performed in triplicate.

Supporting Table S3: pK_d values of the fluorescent MR ligands **6** and **7** from BRET saturation binding experiments in comparison to binding data measured at wild-type M_1 receptors

compound	pK_d (BRET, Nluc-M_1R(Nterm))[a]	N	pK_d (BRET, Nluc-M_1R(G176))[a]	N	pK_i (radioligand comp. binding)[b]
**6**	7.68 ± 0.04	3	8.22 ± 0.03	3	8.21
**7**	8.04 ± 0.04	3	8.60 ± 0.06	3	8.15

[a]Determined by BRET saturation binding experiments at intact HEK293T cells stably expressing the indicated construct. Data are given as means ± SEM of N independent experiments; [b]Gruber et *al.*[5]

Supporting Table S4: pK_d values of **2** and **3** determined in BRET saturation binding experiments at cell homogenates

compound	pK_d (cell homogenates)[a]	*N*	pK_d (intact cells)[b]	*N*
**2**	6.24 ± 0.12	2	8.32 ± 0.08	4
**3**	6.17 ± 0.10	2	8.04 ± 0.08	5

[a]Determined by BRET saturation binding experiments at cell homogenates of HEK293T cells stably expressing Nluc-NTS_1R(T227) (for ligand **2**) or Nluc-AT_1R(S186) (for ligand **3**); [b]determined by BRET saturation binding experiments at intact HEK293T cells stably expressing Nluc-NTS_1R(T227) (for ligand **2**) or Nluc-AT_1R(S186) (for ligand **3**); values were taken from Table 3 in the main article. Data are given as means ± SEM of *N* independent experiments.

Synthesis of the fluorescent angiotensin II derivative UR-AP177 (3)

As outlined in the following scheme, the fluorescent angiotensin II derivative **3** (UR-AP177) was prepared from the recently reported angiotensin II analogue UR-MK302 (**SI1**), containing an amino-functionalized N$^{\omega}$-carbamoylated arginine in position 2,[2] and from the pyrylium dye Py-5 (**SI2**),[6] which was prepared according to a reported procedure.[7]

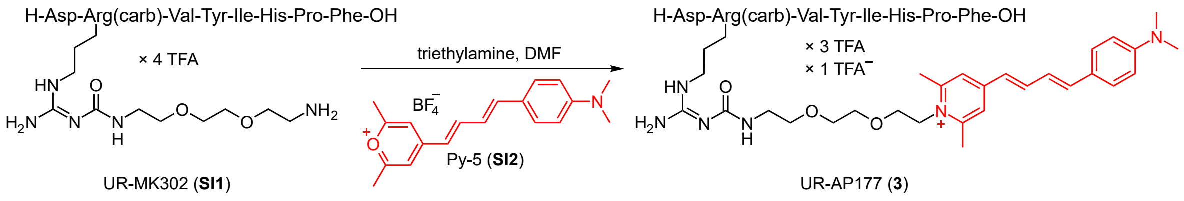

Asp-*N*$^{\omega}$-(*N*-[8-{2,6-Dimethyl-4-(4-[4-{*N*,*N*-dimethyl}aminophenyl]but-1,3-dien-1-yl)pyridin-1-ium-1-yl}-3,6-dioxaoctyl]aminocarbonyl)-Arg-Val-Tyr-Ile-His-Pro-Phe trifluoroacetate tris(hydrotrifluoroacetate) (UR-AP177, 3)

The synthesis of **3** (UR-AP177) was performed in a 1.5-mL polypropylene reaction vessel with screw cap (Süd-Laborbedarf, Gauting, Germany) equipped with a micro stir bar. Amine precursor UR-MK302 (**SI1**, 5.1 mg, 3.0 µmol) was dissolved in anhydrous dimethylformamide (DMF, 300 µL). Triethylamine (4 µL, 30 µmol) was added resulting in a precipitation of the peptide. Therefore, N-methylpyrrolidine (NMP, 300 µL) was added to resolubilize the peptide. A solution of Py-5 (**SI2**, 1.65 mg, 4.5 µmol) in anhydrous DMF/anhydrous NMP 1:1 (v/v) (100 µL) was added and the mixture was stirred at room temperature in the dark for 30 min. Additional Py-5 (**SI2**, 0.55 mg, 1.5 µmol), dissolved in anhydrous DMF/anhydrous NMP 1:1 (25 µL), was added and stirring was continued at rt in the dark for 1 h. 10% aqueous trifluoracetic acid (TFA, 40 µL) was added and the product (UR-AP177, **3**) was isolated by preparative HPLC (system: two K-1800 pumps, a K-2001 detector and a manual injector (Knauer, Berlin, Germany), stationary phase: Kinetex-XB C18, 5 µm, 250 × 21 mm (Phenomenex, Aschaffenburg, Germany), gradient: 0-25 min: 0.1% aqueous TFA/acetonitrile 90:10-45:55, flow: 20 mL/min, column temperature: ambient, detection wavelength: 220 nm, t_R (**3**) = 18 min). Lyophilization of the eluate afforded the product as a dark red fluffy solid (0.74 mg, 13%).

HRMS (Agilent 6540 UHD Accurate-Mass Q-TOF LC/MS system coupled to an Agilent 1290 HPLC system (Agilent Technologies, Santa Clara, CA), ionization: ESI): m/z $[M^{+}+H]^{2+}$ calcd. for $[C_{76}H_{106}N_{16}O_{15}]^{2+}$ 741.4006, found 741.4023. RP-HPLC (system: a 1290 Infinity binary pump equipped with a degasser, a 1290 Infinity Autosampler, a 1290 Infinity Thermostated Column Compartment and a 1260 Infinity Diode Array Detector (Agilent Technologies), stationary phase: Kinetex-XB C18, 2.6 µm, 100 × 3 mm (Phenomenex), gradient: 0-12 min: 0.04% aqueous TFA/acetonitrile 90:10-55:45, 12-16 min: 55:45-5:95, 16-20 min: 5:95 (isocratic), flow rate: 0.5 mL/min, oven temperature: 25 °C, detection wavelength: 220 nm): 96% (t_R = 8.8 min, k = 8.6). $C_{76}H_{105}N_{16}O_{15}^{+} \cdot C_8H_3F_{12}O_8^{-}$ (1482.77 + 455.09).

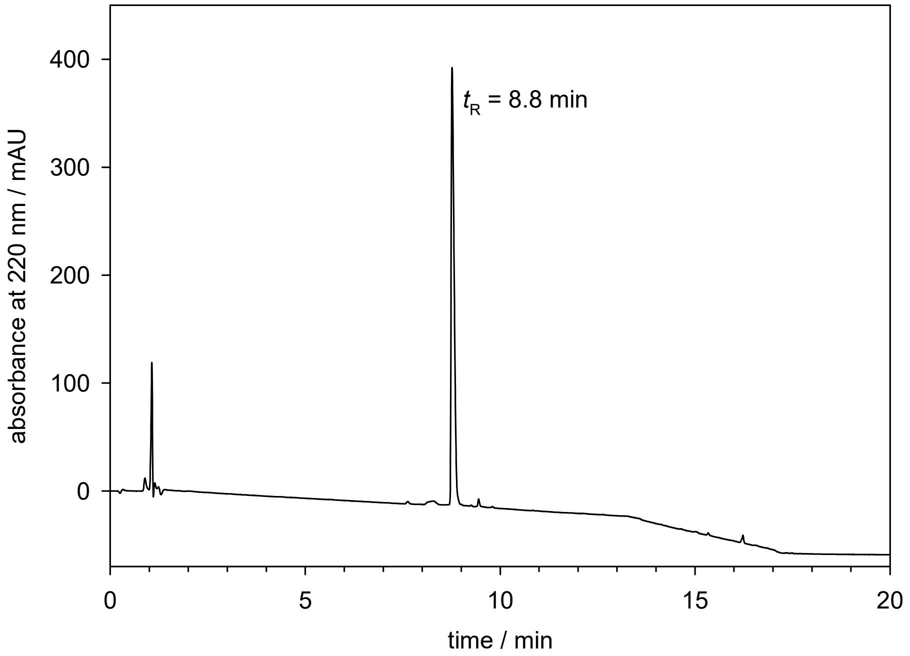

Chromatogram of the RP-HPLC analysis of **3** (UR-AP177).

Synthesis of BIBP3226 (SI5)

The commonly used Y_1R antagonist BIBP3226[8] was prepared from ornithine derivative **SI3** (synthesis described previously[9]) which was guanidinylated by treatment with the pyrazole-type guanidinylating reagent **SI4** (synthesis described elsewhere[10]), followed by removal of the acid-labile protecting groups (Boc groups, phenolic tert-butyl group) using trifluoroacetic acid (see Scheme below).

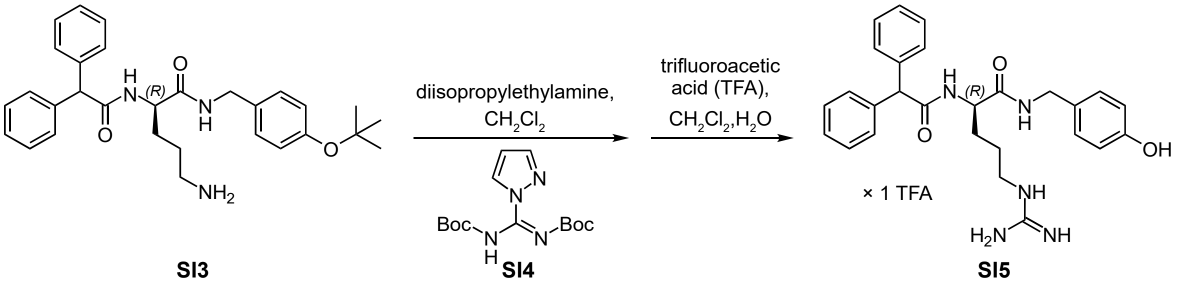

(*R*)-*N*$^{\alpha}$-Diphenylacetyl-(4-hydroxybenzyl)argininamide hydrotrifluoroacetate (SI5). Guanidinylating reagent **SI4** (245 mg, 0.79 mmol) was added to a solution of amine **SI3** (350 mg, 0.72 mmol) and diisopropylethylamine (63 µL, 0.36 mmol) in dichloromethane (6 mL) and the mixture was stirred at room temperature for 1.5 h. The mixture was directly subjected to column chromatography (stationary phase: silica gel Geduran 60, 63-200 µm (Merck), eluent: dichloromethane/ethyl acetate 10:1 to 4:1) to isolate the protected intermediate. The latter was dissolved in dichloromethane (2 mL) followed by the addition of water (0.2 mL) and trifluoroacetic acid (2 mL). The mixture was stirred at room temperature for 2 h, dichloromethane (20 mL) was added and the volatiles were evaporated. The product was purified by reversed-phase flash chromatography using a preparative HPLC system (two K-1800 pumps, a K-2001 detector and a manual injector (Knauer, Berlin, Germany)) and a Puriflash 15C18HQ cartridge (120 g) (Interchim, Montluçon, France) as stationary phase. The following eluent and gradient, respectively, was used: 0-30 min: 0.2% aqueous TFA/acetonitrile 85:15-43:57. The flow rate was 50 mL/min and the detection wavelength was set to 220 nm. Lyophilization of the eluate, containing the product (t_R = 15 min), afforded BIBP3226 (**SI5**) as a white fluffy solid (358 mg, 85%). ^{1}H-NMR (Bruker Avance 600 instrument

with cryogenic probe (Bruker, Karlsruhe, Germany), 600 MHz, [D_6]DMSO): δ (ppm) 1.32-1.46 (m, 2H), 1.49-1.57 (m, 1H), 1.63-1.71 (m, 1H), 3.02-3.10 (m, 2H), 4.10-4.20 (m, 2H), 4.30-4.36 (m, 1H), 5.13 (s, 1H), 6.65-6.70 (m, 2H), ca. 6.95 (br s, 2H, interfering with the next listed signal), 6.98-7.02 (m, 2H), 7.20-7.25 (m, 2H), ca. 7.26 (br s, 2H, interfering with the signal listed before and with the next listed signal), 7.26-7.32 (m, 8H), 7.59 (t, 1H, *J* 5.8 Hz), 8.36 (t, 1H, *J* 5.8 Hz), 8.48 (d, 1H, *J* 8.1 Hz), 9.31 (br s, 1H). ^{13}C-NMR (Bruker Avance 600 instrument with cryogenic probe (Bruker), 150 MHz, [D_6]DMSO): δ (ppm) 25.1, 29.5, 40.3, 41.6, 52.3, 55.9, 115.0, 116.6 (q, *J* 296 Hz) (TFA), 126.5, 126.6, 128.1, 128.2, 128.4, 128.48, 128.50, 129.1, 140.3, 140.5, 156.2, 156.7, 158.5 (q, *J* 33 Hz) (TFA), 170.96, 170.99. HRMS (Agilent 6540 UHD Accurate-Mass Q-TOF LC/MS system coupled to an Agilent 1290 HPLC system (Agilent Technologies, Santa Clara, CA), ionization: ESI): *m/z* [M+H]$^+$ calcd. for [$C_{27}H_{32}N_5O_3$]$^+$ 474.2500, found 474.2505. RP-HPLC (system: a SN400 controller, a P4000 pump, an AS3000 autosampler and a Spectra Focus UV−vis detector (Thermo Separation Products) as well as a degasser Degassex DG-4400 (Phenomenex), stationary phase: Eurospher-100 C18, 5 µm, 250 × 4 mm (Knauer, Berlin, Germany), gradient: 0-30 min: 0.05% aqueous TFA/acetonitrile 80:20-5:95, 30-40 min: 5:95 (isocratic), flow rate: 0.8 mL/min, oven temperature: 30 °C, detection wavelength: 220 nm): 99% (t_R = 12.2 min, k = 3.5). $C_{27}H_{31}N_5O_3 \cdot C_2HF_3O_2$ (473.24 + 114.02).

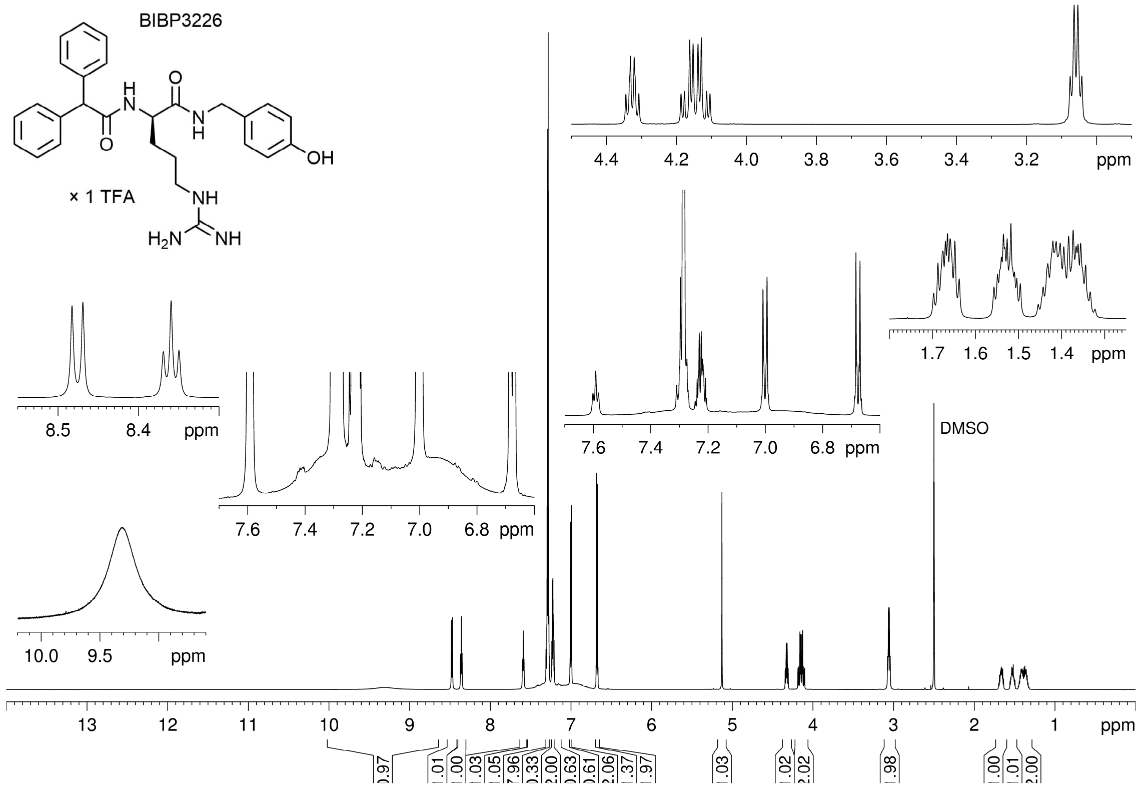

^{1}H-NMR spectrum (600 MHz, [D$_6$]DMSO) of BIBP3226 (**SI5**)

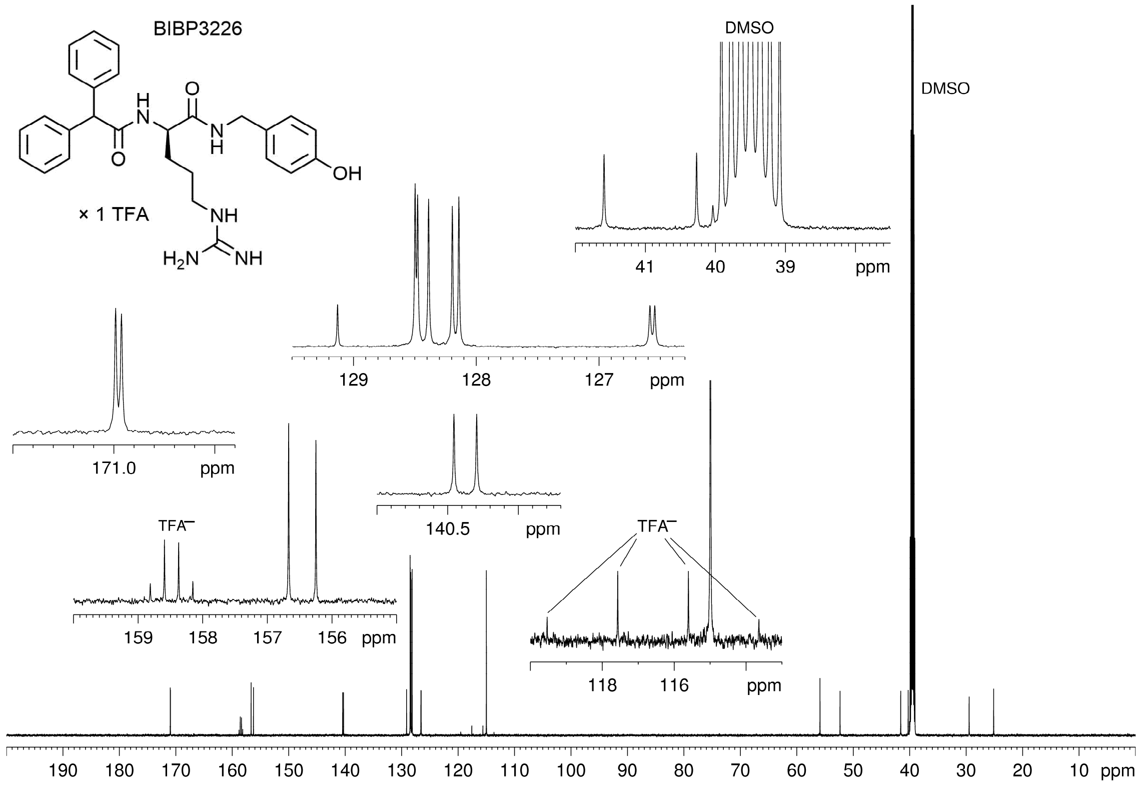

^{13}C-NMR spectrum (150 MHz, [D$_6$]DMSO) of BIBP3226 (**SI5**)

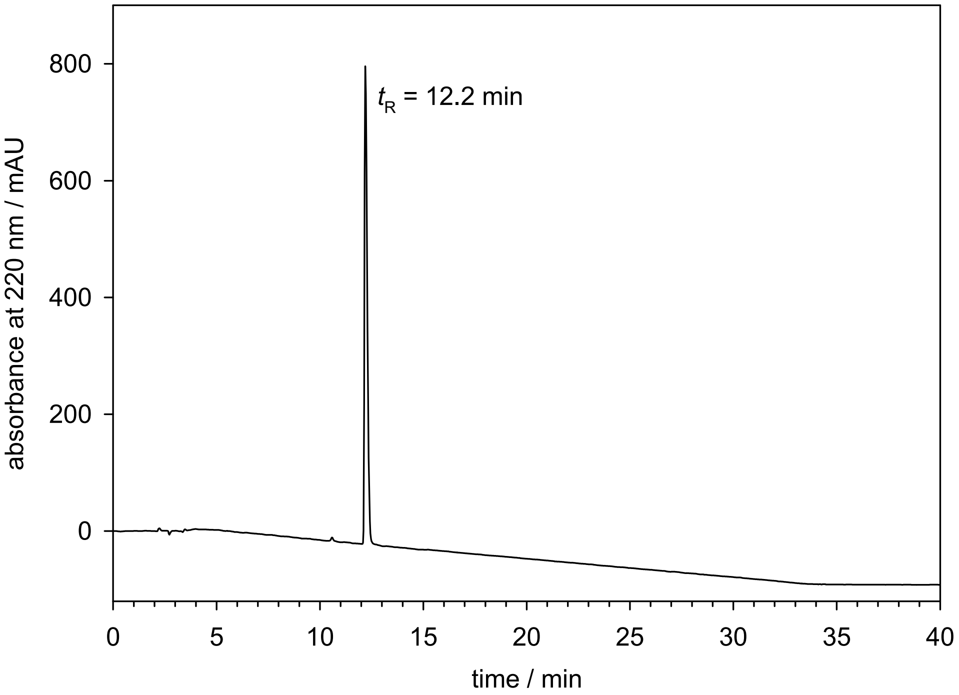

Chromatogram of the RP-HPLC analysis (purity control) of BIBP3226 (**SI5**)

References

1. Keller, M.; Weiss, S.; Hutzler, C.; Kuhn, K. K.; Mollereau, C.; Dukorn, S.; Schindler, L.; Bernhardt, G.; König, B.; Buschauer, A., N^{ω}-Carbamoylation of the argininamide moiety: an avenue to insurmountable NPY Y_1 receptor antagonists and a radiolabeled selective high-affinity molecular tool ([^{3}H]UR-MK299) with extended residence time. *J. Med. Chem.* **2015,** *58* (22), 8834-8849.

2. Keller, M.; Kuhn, K. K.; Einsiedel, J.; Hübner, H.; Biselli, S.; Mollereau, C.; Wifling, D.; Svobodová, J.; Bernhardt, G.; Cabrele, C.; Vanderheyden, P. M. L.; Gmeiner, P.; Buschauer, A., Mimicking of arginine by functionalized N^{ω}-carbamoylated arginine as a new broadly applicable approach to labeled bioactive peptides: high affinity angiotensin, neuropeptide Y, neuropeptide FF, and neurotensin receptor ligands as examples. *J. Med. Chem.* **2016,** *59* (5), 1925-1945.

3. Isberg, V.; Mordalski, S.; Munk, C.; Rataj, K.; Harpsøe, K.; Hauser, A. S.; Vroling, B.; Bojarski, A. J.; Vriend, G.; Gloriam, D. E., GPCRdb: an information system for G protein-coupled receptors. *Nucleic Acids Res.* **2015,** *44* (D1), D356-D364.

4. Kooistra, A. J.; Mordalski, S.; Pándy-Szekeres, G.; Esguerra, M.; Mamyrbekov, A.; Munk, C.; Keserű, G. M.; Gloriam, David E., GPCRdb in 2021: integrating GPCR sequence, structure and function. *Nucleic Acids Res.* **2020**, gkaa1080.

5. Gruber, C. G.; Pegoli, A.; Müller, C.; Grätz, L.; She, X.; Keller, M., Differently fluorescence-labelled dibenzodiazepinone-type muscarinic acetylcholine receptor ligands with high M_2R affinity. *RSC Med. Chem.* **2020,** *11* (7), 823-832.

6. Wetzl, B. K.; Yarmoluk, S. M.; Craig, D. B.; Wolfbeis, O. S., Chameleon labels for staining and quantifying proteins. *Angew. Chem. Int. Ed. Engl.* **2004,** *43* (40), 5400-5402.

7. Höfelschweiger, B. K. The pyrylium dyes: a new class of biolabels. Synthesis, spectroscopy, and application as labels and in general protein assay. PhD Thesis, University of Regensburg, 2005.

8. Rudolf, K.; Eberlein, W.; Engel, W.; Wieland, H. A.; Willim, K. D.; Entzeroth, M.; Wienen, W.; Beck-Sickinger, A. G.; Doods, H. N., The first highly potent and selective non-peptide neuropeptide Y Y_1 receptor antagonist: BIBP3226. *Eur. J. Pharmacol.* **1994,** *271* (2), R11-R13.

9. Keller, M.; Pop, N.; Hutzler, C.; Beck-Sickinger, A. G.; Bernhardt, G.; Buschauer, A., Guanidine−acylguanidine bioisosteric approach in the design of radioligands: synthesis of a tritium-labeled N^G-propionylargininamide ([^{3}H]-UR-MK114) as a highly potent and selective neuropeptide Y Y_1 receptor antagonist. *J. Med. Chem.* **2008,** *51* (24), 8168-8172.

10. Brennauer, A. Acylguanidines as bioisosteric groups in argininamide-type neuropeptide Y Y_1 and Y_2 receptor antagonists: synthesis, stability and pharmacological activity. PhD Thesis, University of Regensburg, 2006.
